# Communicating Risk in the Age of Misinformation: Empirical Frameworks for Understanding Stakeholder Trust

**DOI:** 10.1111/risa.70337

**Published:** 2026-08-21

**Authors:** Matthew S. Weber, Chaeyeong Margo Lee, David S. Kosson

**Affiliations:** ^1^ Department of Communication, School of Communication and Information Rutgers University New Brunswick New Jersey USA; ^2^ Department of Civil and Environmental Engineering, School of Engineering Vanderbilt University Nashville Tennessee USA

**Keywords:** complex information, misinformation, systematic review, trust

## Abstract

This systematic literature review examines how stakeholder trust is conceptualized and measured in risk and science communication amid increasingly complex information environments shaped by misinformation and disinformation. Following PRISMA procedures, which provide a framework for conducting a systematic review with transparency, replicability, and completeness, we searched major databases and screened studies using explicit inclusion criteria. For instance, trust had to be operationalized and measured in risk or scientifically complex contexts; credibility studies were retained when credibility was a trust‐related dimension related to stakeholder communication. Scientifically complex contexts are taken to refer to settings where information is inherently complex and nuanced, and understanding requires advanced knowledge. The final corpus (*k* = 69) includes quantitative, qualitative, and mixed‐methods designs, with a post‐2020 increase aligning with pandemic‐era scholarship. The findings identify three roles for trust. First, stakeholder trust as an outcome is strengthened more by transparency, empathy, and perceived value similarity than by expertise claims alone. Second, trust as a broad concept often embodies more than just detailed technical understanding; rather, trust predicts acceptance of technologies and policies and lower perceived risk. Third, as a mechanism, stakeholder trust mediates links between credibility, transparency, or value similarity and downstream outcomes such as compliance and cooperation. From organizations to interpersonal interactions, stakeholder trust formation reflects the characteristics of trustors, trustees, and context. We integrate these strands into a communication approach that emphasizes clarity about uncertainty, engagement, and visible accountability as preconditions for trust. Practically, the review underscores the design of transparent messaging, alignment with public values without oversimplification, and participatory approaches that treat stakeholders as partners. Conceptually, we separate judgments about whether information seems credible from trust in the people or institutions behind it. We argue that trust helps connect what people think and feel to what they are willing to do, especially when they must make decisions under uncertainty in today's complex information environment.

## 1 Introduction

In an era defined by information abundance and uncertainty, institutions face growing challenges in maintaining trust while communicating about science, technology, and risk. The proliferation of digital media has transformed how people encounter, interpret, and evaluate knowledge, amplifying both the reach of expertise and the spread of misinformation (Caled and Silva 2022). Against this backdrop, the ability to effectively communicate complex or uncertain scientific information is a defining concern for policymakers, communicators, and researchers alike. Clearly, communicating complex knowledge on high‐risk scientific issues such as radioactive waste, where the public perceives high risk to human health and the environment from potential mismanagement, remains a persistent challenge for institutions. Similar issues exist in contexts such as global pandemics, where complex information must be relayed about transmission and treatment, as well as cases of drinking water contamination, where institutions must communicate uncertain evidence about exposure, long‐term health effects, and remediation while addressing public distrust and rapidly circulating misinformation. This is especially complicated where misinformation can quickly distort public understanding. Broader contexts with perceived high risk include a variety of topics, such as information about food safety and medical information, among others. Clear and effective communication of scientific information is an ever‐present challenge in the modern era; the proliferation of disinformation is a notable risk to effectively communicate accurate information, especially as foreign adversaries seek to weaponize information against critical networks (Barker et al. 2025).

Researchers have long sought to distinguish between scientific misinformation, disinformation, and broader issues of stakeholder trust in information. Within this context, stakeholder trust is both a prerequisite for and outcome of effective communication, particularly when communicating complex scientific knowledge. Scholars of risk communication have emphasized that building and maintaining trust with key stakeholders remains a critical challenge, especially when information is contested or evolving (Elisabetta Galeotti and Meini 2022). In risk communication, stakeholders are any individual, group, or organization that can affect, be affected by, or perceive themselves to be affected by a risk or the decisions made to manage (Ndlela 2018). This systematic literature review focuses on the core question of what is known from an empirical perspective about the relationship between perceptions of trust in risk and stakeholder engagement and perceptions of risk in the context of complex information landscapes. Risk communication is a powerful tool for building trust with key stakeholders during periods of uncertainty and when information being shared with stakeholders is complex or contested. In recent years, this type of communication has been critical in ongoing efforts to establish trusted pathways of communication in response to the rapid proliferation of misinformation and disinformation (Balog‐Way and McComas 2025).

Accordingly, this article reviews empirical research examining how stakeholder trust is defined, operationalized, and measured within risk and science communication. Drawing on 69 studies, this article maps stakeholder trust, identifies methodological trends, and evaluates the communicative mechanisms through which trust is built or eroded in complex information environments. A complex information environment is taken to be an interconnected network of media entities and consumers, blending traditional information sources (word of mouth, radio, television stations, etc.) as well as user‐generated content and social media platforms. The findings emphasize three key themes: trust as an outcome, trust and resulting behaviors, and trust as a mechanism.

## 2 Core Constructs and Key Definitions

Trust is “a state in which one person (trustor) chooses to rely on another person (trustee) in a risky situation based upon positive expectations of the trustee's behavior or intentions” (Rousseau et al. 1998, as cited in Conchie and Burns 2009, 14). More specifically, thinking about how information is communicated by organizations, “trust in communication refers to the generalized expectancy that a message received is true and reliable and that the communicator demonstrates competence and honesty by conveying accurate, objective, and complete information” (Renn and Levine 1991, 179). Misinformation is defined as false or misleading information presented as fact without malicious intentions (Jennings and Stroud 2023). Misinformation is distinct from disinformation, which is purposefully generated for financial gains or to cause harm politically and personally (Buchanan and Benson 2019; Swire‐Thompson and Lazer 2020) and has received significant attention in the 2020s.

This study examines how levels of stakeholder trust shape public interpretation of information and how communication from trusted entities can influence belief formation and attitude change. There has been an expansion of research regarding policy and research to combat misinformation, but Aven and Thekdi (2022) note that this work often oversimplifies communication by focusing on the content of the message as opposed to understanding the impact of the sender and receiver. Indeed, echoing this sentiment, we argue that there is a need to better understand how risk and uncertainty are communicated to stakeholders when facing complex information landscapes. Risk communication has long established the importance of the perceived trustworthiness of messengers, but given the growth of scholarship in the past decade, a closer examination is warranted.

### 2.1 Misinformation and Complex Information

When communicating to stakeholders, there is an increased reliance on intermediaries such as scientists, regulators, and journalists. These stakeholders are critical in communicating about risk. Furthermore, these stakeholders must be trusted and establish their trustworthiness to others (O'Neill 2018). Admittedly, it is difficult to unpack the intent behind the creation and sharing of information (Li and Santos Jr. 2020). In addition, it becomes more challenging in the online environment as information circulates and is decontextualized, undergoing substantial transformation that obscures both its original source and the creator's intent (Hameleers 2023; Shamayleh and Arsel 2026). Rather than disentangling intention or tracing the original provenance of content, this centers on sources through which information is disseminated and the factors that influence why certain sources are perceived as more trustworthy than others.

At the individual level, characteristics of the trustor—including their propensity to trust, past experiences, cognitive capacity, and scientific literacy—are significant factors (van Antwerpen et al. 2025). Individuals who are more scientifically literate are generally better at detecting credibility cues such as expertise and vested interest, while those with a high baseline propensity to trust may still revise their judgments based on negative past experiences. Expertise is a product of social interactions rather than simply being a property of an individual through cognitive skill or professional standing (Treem 2012). A vested interest refers to a stakeholder's personal or organizational stake in the outcome of a situation; moreover, sociocultural and political orientations significantly influence which institutions and figures are perceived as credible. For example, individuals are more inclined to trust information from sources that align with their political identity or cultural values, and self‐assessed knowledge can further mediate the relationship between trust and concern—particularly when filtered through attitudes toward scientists or institutions (Malka et al. 2009; Vainio et al. 2017).

Equally important are the attributes of the trustee or the source being evaluated. Perceptions of expertise, reputation, and integrity are central to judgments of trustworthiness (Hancock et al. 2023; van Antwerpen et al. 2025). Sources perceived as knowledgeable and reliable are more likely to be trusted, particularly when they are unaffiliated with institutions that carry overt vested interests. For example, academic researchers and public institutions tend to be rated as more credible than private industry actors or lobbyists. Transparency, performance history, and interpersonal characteristics—such as consistency and fairness—also enhance credibility. While cues related to expertise are generally strong predictors of trust, public sensitivity to conflicts of interest may be more variable, particularly in less informed audiences.

Finally, contextual factors—such as group membership, communication dynamics, and environmental complexity—further shape trust relationships. Trust increases when communication is clear and effective, shared mental models exist, and the perceived relationship between parties is close or cooperative (Hancock et al. 2023). In‐group affiliation often amplifies initial trust, even when objective indicators of credibility are weak. In high‐risk or high‐uncertainty environments, such as those involving environmental or scientific risk communication, these contextual variables become even more influential. The digital information ecosystem introduces additional complexity: social media platforms facilitate rapid information spread but also foster epistemic bubbles and echo chambers (Kirmayer 2024). This may include a distortion of and loss of provenance of the original information. Perceived value similarity between the audience and the source further determines credibility—organizations seen as sharing the public's values are more likely to be trusted, even when objective competence may be contested (Vainio et al. 2017). Trust is not only a function of facts or authority but also deeply embedded in individual identity and context. These factors underscore the need for systematic synthesis of empirical research examining how trust is operationalized and measured across diverse risk communication contexts.

## 3 Methods and Data

### 3.1 Procedure

**FIGURE 1:**
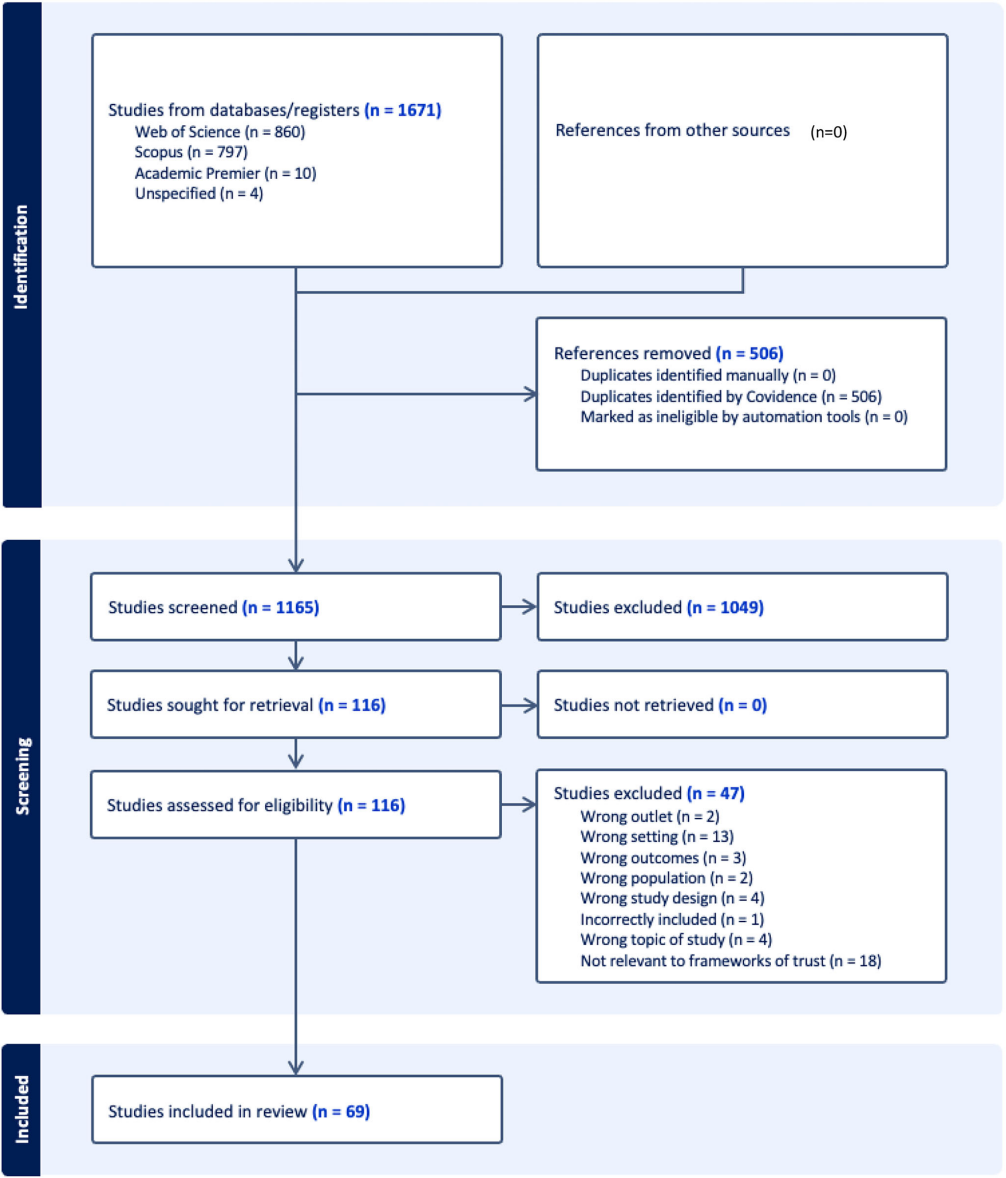
PRISMA flowchart of the article selection process.

This review was conducted in accordance with the Preferred Reporting Items for Systematic Reviews and Meta‐Analyses (PRISMA) guidelines (Page et al. 2021), mirroring similar approaches examining risk contexts via a systematic review (i.e., Schuster and Scheu 2026). A PRISMA flow diagram outlining the literature search and screening process is presented in Figure 1. The goal was to identify, evaluate, and synthesize existing research on frameworks for conceptualizing stakeholder trust in information and institutions, especially in the context of complex information landscapes.

### 3.2 Inclusion/Exclusion Criteria

In conducting this systematic review, we did not make an a priori decision regarding the causal relationship between stakeholder trust and information. Information is taken to be misinformation‐related content, message themes, framing, and knowledge shared via media or interpersonal communication. Furthermore, we did not explicitly distinguish between stakeholder trust in information and disinformation/misinformation but treated these different variables collectively as distinct types of information. We focus on a broad array of contexts to better understand the different ways in which stakeholder trust in information is conceptualized in risk communication research. For an in‐depth discussion of this criterion, please refer to Appendix 1 in Supporting Information.

In summary, the following criteria were applied:

- An article needed to be an empirical research article; we included both qualitative and quantitative approaches.
- Included articles conceptualized risk communication and information in the context of trust, with an emphasis on understanding the mediating and moderating variables. We emphasized studies where risk was a focal element of the research; risk conversations or the communication of risk was a key dimension of the study.
- Trust needed to be explicitly operationalized and empirically measured rather than assumed or inferred. Articles that treat trust only as a background or contextual descriptor were therefore excluded.
- Studies addressing credibility are included only when credibility functions as a trust‐related dimension, such as trustworthiness, integrity, or benevolence, contributing to the formation or reinforcement of trust in communicators or institutions.
- An article needed to examine communication in a risk‐involved or scientifically complex context characterized by uncertainty, potential harm, and institutional responsibility (e.g., environmental hazards, public health, safety, or science communication).
- Articles from consumer, marketing, branding, or technological contexts (e.g., cybersecurity, blockchain, and cloud computing) were excluded when trust was conceptualized as transactional or functional—focused on customer loyalty, satisfaction, system reliability, or performance assurance measures—rather than as a relational or communicative construct.

### 3.3 Search Strategy

An informal review was conducted to initially identify the most pertinent keywords and to create a guideline for the terminology included in this study. The research team reviewed and discussed 15 relevant articles and selected terminology from those articles to guide the search.

To identify, evaluate, and synthesize research on trust‐related variables and frameworks, we executed a structured database search and a two‐stage screening process. A comprehensive search was conducted across multiple electronic databases, including Web of Science, Academic Search Premier, and SCOPUS. The primary search used a Topic (TS) query designed to capture studies at the intersection of four key domains: trust, credibility, risk or science communication, and related frameworks or models. The search strings are provided in the appendix for replication (see Appendix 2 in Supporting Information for search queries). We focused on the inclusion of recent theoretical advancements and contemporary empirical work, but we did not explicitly limit the time frame. Indeed, because of the search terms, there was an inherent surge in relevant research between 2015 and 2025.

### 3.4 Screening Process

After removing duplicates, the remaining articles were screened in two stages: Title and abstract review followed by a full‐text review. PRISMA calls for the elimination of articles that cannot be retrieved, but that was not an issue with this analysis. During the first round of screening, which resulted in 1165 articles, the primary focus was to review each article to assess the quality of the journal in which it was published (generally based on SCOPUS ratings to ensure Q1 and Q2 journals), evaluate the extent to which the study's outcomes aligned with our focus on frameworks for stakeholder trust, and determine the relevance of the population under study based on information provided in the abstract. This process allowed us to quickly identify studies that did not substantively address trust or credibility in the context of information and institutions or that examined populations outside the scope of our review. For instance, studies situated in contexts such as energy, disaster or crisis management, health, and environmental issues—particularly those involving waste, contamination, and remediation—were considered relevant and retained. In contrast, studies situated in domains such as marketing, consumer behavior, retailing, cybersecurity, and privacy technologies were excluded and outside the scope of the review (*k* = 13), as they focused on trust in a specific business or application context without a general model of trust or theory of trust. Similarly, studies focused on a critical cultural analysis of trust and information were also excluded (*k* = 7).

The second stage of screening involved a full‐text review of 116 articles that had passed the initial stage. In this phase, we applied our inclusion and exclusion criteria in greater detail, with attention to distinguishing between studies that treated trust merely as an ancillary variable—often operationalized in terms of source credibility or perceived reliability of information in digital environments—and those in which trust functioned as a central conceptual or theoretical focus. Specifically, we retained studies if they conceptualized trust as a core construct, explored its role as a mediating or moderating factor, or examined it as a primary outcome within a theoretical framework related to science or risk communication. In contrast, studies that framed trust tangentially, such as in the evaluation of message clarity, source credibility, or the effectiveness of information delivery, without situating these elements within a broader framework for understanding trust were excluded.

## 4 Findings

### 4.1 Corpus Composition: Citations and Methodological Makeup

The finalized corpus comprised 69 studies, spanning publication years from 1997 to 2025. The full list of articles included in the review is provided as an appendix (see Appendix 3 in Supporting Information). The temporal distribution indicates a marked increase in publications after 2020, coinciding with the rise of COVID‐19‐related research on trust, risk communication, and online information behaviors. This surge aligns with trends observed in related disciplines emphasizing misinformation, science communication, and institutional trust (see Phogat et al. 2025 for a detailed analysis of the research surge following the peak of the COVID‐19 pandemic), as well as an increasing reliance on digital information.

In terms of citation makeup, several texts appear as methodological or conceptual anchors across the dataset, including Siegrist and Cvetkovich (2000), Frewer et al. (2003), and Trumbo and McComas (2003). The average citation count for articles in this analysis was 137 citations per article; the three preceding references received 1315, 617, and 284 citations, respectively, as of April 2026, clearly pointing to their centrality in this review. Siegrist and Cvetkovich's (2000) introduction of the concept that value similarity between the public and institutions fosters social trust, which in turn influences risk and benefit perceptions for technological and environmental hazards, was frequently used to justify the inclusion of trust as a mediating variable and to ground quantitative modeling of trust‐risk dynamics in contexts such as nuclear energy and food safety. Similarly, Frewer et al. (2003) is foundational in the corpus for studies analyzing risk communication, as it provides both the theoretical rationale for source credibility effects—such as trust in source expertise and intentions—and an empirical template for modeling trust as a mediator between message exposure and attitudinal outcomes. In addition, Trumbo and McComas's (2003) research on the role of credibility assessments in shaping risk perceptions regarding cancer cluster investigations demonstrated that higher citizen group credibility increased risk perception, while state and industrial credibility had the opposite effect. This work was cited as a key empirical touchstone for structuring comparative analyses of source credibility, as well as modeling the direct and indirect effects of credibility on risk perception in public health and environmental domains. Recent works, such as Alabri (2025) and Zhang et al. (2025), signal renewed interest in cognitive and behavioral responses to trust in institutions and media to information reliability in technologically mediated (social media, AI‐generated content, etc.) contexts. These recurring references reveal continuity between classic models of trust and contemporary theories linking misinformation to social cognition and media reliance.

Methodologically, there is a split of 43 quantitative articles and 26 qualitative articles. Of the 43 quantitative articles, 33 use cross‐sectional surveys, 3 use simulations, 5 are experimental, 1 uses mixed methods, and 1 is longitudinal. Quantitative studies typically used regression or structural equation modeling to test associations between trust, risk perception, and behavioral intention. The qualitative articles rely on interviews and focus groups (nine) and textual or content analysis (two), while the remaining are conceptual and/or theoretical and specifically review or address empirical frameworks of trust. This qualitative work explored discursive constructions of trust but also included relevant textual analyses and other systematic reviews that addressed aspects of empirical measurement. The methodological diversity of scholarship in this space supports a triangulated understanding of how trust operates across communication domains and risk contexts. While the blend of quantitative and qualitative research may not explicitly reinforce causal pathways, it does help to advance an understanding of how trust is conceptualized, experienced, or situated across contexts.

In terms of citations, *Proceedings of the National Academy of Sciences* had the most citations per article (682 citations), but there was only a single article in the dataset from that journal. Looking at journals that had more than three articles in the dataset, *Risk Analysis* had the most articles per journal (*n* = 9) and an average of 119 citations per article. *Journal of Risk Research* averaged 95 citations per article (*n* = 8), and *Science Communication* averaged 88 citations per article (*n* = 3). The full breakdown of journals and journal topics is provided in Appendix 4 in Supporting Information.

### 4.2 Key Concepts Operationalized in Extant Literature

In the reviewed literature, trust is conceptualized as a psychological and relational construct that reflects an individual's willingness to rely on other actors—such as an institution, expert, or system—under conditions of uncertainty and vulnerability (where vulnerability indicates that an individual is susceptible to information of varying degrees of reliability). It is commonly defined as the expectation that the trusted party possesses competence, honesty, and benevolence (Peters et al. 1997), as appears in six articles among the quantitative studies. Within communication and risk research, trust extends beyond interpersonal dynamics to encompass stakeholder trust or confidence in the fairness, transparency, and accountability of organizations that manage or disseminate information (Cvetkovich and Nakayachi 2007; Siegrist et al. 2000).

Importantly, trust is defined differently depending on the context. In risk‐involved situations—such as environmental hazards, occupational safety, or communication of sensitive information—trust often entails an active choice to accept vulnerability. Rousseau et al. (1998, 385) describe it as “a state in which one person (trustor) chooses to rely on another person (trustee) in a risky situation based upon positive expectations of the trustee's behavior or intentions.” For example, Conchie and Burns (2009) applied this framing to occupational safety, illustrating that employees' trust in management was grounded in perceptions of shared risk and moral responsibility, not simply managerial competence. Likewise, in environmental or health risk contexts, trust involves the anticipation of reliable, ethical, and value‐aligned actions from institutions responsible for protecting public welfare.

Operationally, studies measure trust across three core dimensions: Competence, referring to belief in an actor's expertise or capability (Peters et al. 1997; White and Johnson 2010); integrity, or perceived honesty and consistency in communication (Nakayachi and Ozaki 2014); and benevolence, the belief that the actor acts with concern for the public interest (Conchie and Burns 2009; Ryu et al. 2018). In mediated and scientific contexts, trust often overlaps with credibility judgments, encompassing evaluations of reliability, accuracy, and openness that influence whether audiences perceive information as legitimate (Besley et al. 2012; Bigham et al. 2019; Lederman et al. 2014; Trumbo and McComas 2003). This includes an evaluation of the information shared as well as the way in which the information is communicated (the type of terminology, the complexity of the language, etc.). Collectively, these definitions frame trust as both a cognitive assessment and affective orientation, forming the foundation for cooperative behavior, information acceptance, and legitimacy in the communication of complex or risk‐laden knowledge (Myrick and Hendryx 2021; Seebauer and Babcicky 2018; Wilkins et al. 2018; Yi et al. 2013).

### 4.3 The Role of Trust as a Critical Outcome in Understanding Complex Information

Across the reviewed studies, a dominant line of inquiry conceptualizes trust as an outcome, examining the factors that build or diminish trust in institutions, communicators, and systems managing complex or risk‐laden information. Consistently, message transparency, perceived honesty, competence, and benevolence emerged as central predictors of trust. For instance, Peters et al. (1997) found that honesty and openness were stronger determinants of trust than technical expertise alone. Similar findings were reported in Wilson et al. (2017), who observed that empathetic and transparent communication following the Fukushima disaster increased public confidence in science, while purely factual communication failed to rebuild trust.

Corritore et al. (2012) extended this notion to digital contexts, demonstrating that website credibility—operationalized through honesty and ease of use—directly increased users' trust in online systems. In parallel, White and Johnson (2010) proposed the Intuitive Detection Theorist (IDT) model, showing that perceived competence, cautious decision bias, and openness jointly predict trust in hazard managers, with transparency moderating how errors are judged. Other studies such as Nakayachi and Ozaki (2014) and Peterson et al. (2020) emphasized the role of value similarity and shared risk—individuals trusted managers or institutions more when they perceived them as voluntarily sharing the same risks or values.

In media and public communication, credibility and channel selection also shaped trust outcomes. Wilkins et al. (2018) further observed that scientific organizations and universities were among the most trusted information sources, while governments and private industry ranked lowest. Meanwhile, Erisen and Erisen (2025) and Kožuh and Čakš (2021) demonstrated how political ideology, populist attitudes, and misinformation exposure erode trust in experts and media outlets. In health communication, multiple studies (Latkin et al. 2021; Myrick and Hendryx 2021; Song et al. 2024) consistently found that medical professionals and interpersonal sources generate the highest trust levels, while social media and mass media receive the least. Collectively, these studies illustrate that trust as a dependent variable is driven less by information volume or expertise and more by relational and affective factors—transparency, empathy, and perceived shared values.

### 4.4 Trust as a Catalyst for Communicative Outcomes

Another set of studies conceptualizes trust as a predictor, primarily functioning as a catalyst for behavioral, perceptual, and communicative outcomes. Across these studies, three main patterns emerge: (1) trust facilitates compliance and protective behaviors, (2) it reduces perceived risk and increases acceptance of technologies or policies, and (3) it shapes cognitive and affective processes involved in information seeking and evaluation.

In health and crisis communication contexts, institutional or interpersonal trust consistently predicts compliance with preventive and protective actions. Higher trust in government and public health agencies leads to greater engagement in behaviors such as mask‐wearing, vaccination, and information seeking during disease outbreaks, such as COVID‐19 (Besley et al. 2012; Jin et al. 2021; Tetteh et al. 2022). Likewise, trust in scientific and medical authorities mitigates fear and misinformation, while simultaneously encouraging confidence in institutional guidance (Latkin et al. 2021; Wang et al. 2022). These studies emphasize that trust acts as a motivational resource, enabling the public to convert belief in authority into preventive behavior even amid uncertainty.

Moreover, in technology‐ and environment‐related risk domains, trust reduces perceived threats and fosters acceptance of complex or high‐risk technologies (including contexts as varied as artificial intelligence, nuclear energy, and emerging medical breakthroughs). Trust in regulatory and energy institutions, for example, predicts lower perceived risk and greater acceptance of nuclear power or environmental policy measures (Fujita 2025; Ryu et al. 2018; Vainio et al. 2017). Correspondingly, trust in workplace authorities enhances compliance with safety practices (Conchie and Burns 2009), while trust in local institutions and volunteers encourages civic cooperation in flood prevention (Seebauer and Babcicky 2018). Overall, these studies reveal that trust substitutes for direct technical understanding—when actors are trusted to manage uncertainty, individuals feel less personal need to evaluate risk themselves.

Finally, trust interacts with cognitive and affective mediators such as self‐efficacy, risk perception, and information sufficiency. Ter Huurne and Gutteling (2009) found that both institutional trust and self‐efficacy reduce anxiety and enhance perceived knowledge adequacy, while Wang et al. (2021) demonstrated that trust indirectly promotes protective behavior through increased accuracy of risk perception and information seeking. Moreover, Katsuya (2002) suggested that the effect of trust is conditional on citizens' motivation and prior knowledge, implying that trust's influence is strongest when individuals lack personal expertise.

Framed in a slightly different way, there is also ample evidence from a qualitative perspective that trust is coproduced by communicators and stakeholders through patterns of networked interaction and engagement (*k* = 3). Trust is not merely perceived or evaluated by stakeholders but is actively coproduced through the processes of engagement, communication, and interaction between stakeholders and institutions. Gilmour et al. (2011) provided one of the clearest demonstrations of this notion, showing that participatory design, stakeholder mapping, and mental model interviews helped build trust and foster genuine engagement around biosecurity risk communication. Further evidence shows that when stakeholders are empowered to participate directly in decision‐making, trust shifts from confidence in authorities to confidence in a process stakeholders help to co‐own (Bier 2001). Shahbazi and Bunker (2024) further demonstrated that trust mediates the relationship between the communication of information and stakeholders, with communication functioning to either strengthen or erode trust. Taken together, this line of work suggests that trust is not simply held by stakeholders but is produced through participatory structures, communicative reciprocity, and networked interaction.

Taken together, these findings demonstrate that trust operates as a psychological shortcut that enables cooperation, reduces complexity, and supports decision‐making in uncertain or risk‐laden contexts. Whether promoting health behaviors, technological acceptance, or environmental preparedness, trust consistently serves as a behavioral and perceptual facilitator—bridging the gap between institutional communication and individual action.

### 4.5 Mediators and Moderators of Trust

A smaller subset of studies in this review examined trust as a mediator or moderator. In fact, trust appeared far more often as a mediator (*k* = 7) than as a moderator (*k* = 1), underscoring its role as a relational mechanism that connects cognitive, affective, and behavioral processes in risk and science communication. Only one study (Lee and Jeong 2024) explicitly conceptualized trust as a moderator, showing that in‐group trust amplified the relationship between misinformation belief and social distrust. In other words, when misinformation was shared within a tightly connected group, there was a greater likelihood of group members believing that information. This suggests that tightly bonded trust networks may have strong internal cohesion while inadvertently deepening the proliferation of misinformation. In contrast, at least seven studies (*k* = 7) positioned trust as a mediator, highlighting its consistent function as a psychological bridge between perceptions of credibility, transparency, or value similarity and subsequent outcomes such as risk perception, acceptance, or compliance. A first cluster of studies—primarily in health and crisis communication—found that trust in institutions and media mediated the effect of information seeking and credibility perceptions on emotional and behavioral responses. For instance, Alabri (2025), Guo and Bai (2023), and Peterson et al. (2020) collectively show that individuals who perceive health or media sources as credible are more likely to develop institutional trust, which in turn reduces anxiety and enhances adherence to recommended behaviors.

Next, another group of studies in technology and environmental risk contexts emphasized trust as the channel through which source credibility and transparency influence risk perception and acceptance. Ryu et al. (2018) demonstrated that the credibility of nuclear regulators increased institutional trust, which then reduced perceived risk and strengthened policy support. Similarly, Siegrist et al. (2000) found that social trust rooted in value similarity mediated risk–benefit evaluations of technologies. Together, these findings confirm that trust transforms perceptions of institutional competence and fairness into acceptance and cooperation.

From a qualitative perspective, scholars tended to focus on decomposing trust into grounded dimensions, revealing the component structure of stakeholder trust from the perspective of trustors. Rubin (2012) demonstrated that stakeholders assess motives (e.g., whether information is withheld to prevent panic or for political gain), the transparency of communicators about uncertainty, and the presence or absence of unsubstantiated reassurance to show how stakeholders assess information credibility. Thus, perceived communicator motives shape stakeholders' trust perceptions. Furthermore, expertise, objectivity, work ethic, and potential conflicting interests may also moderate perceptions of trust (Hendriks et al. 2016). Indeed, several qualitative studies in this review (*k* = 5) focused on aspects that moderated perceptions of trust.

As such, these patterns indicate that trust operates less as a conditional factor and more as a communicative process—the affective and cognitive link through which individuals translate credibility and transparency into confidence and action. While moderation effects remain rare, the widespread use of trust as a mediator emphasizes its central role in mobilizing belief, reducing uncertainty, and facilitating engagement in risk and science communication.

### 4.6 Theoretical Constructs Guiding Trust and Information Studies

Although this review focuses explicitly on trust, several included studies measured credibility using trust‐related components, warranting their inclusion due to the conceptual and operational overlap between the two constructs. These studies treat credibility as a multidimensional perception of honesty, expertise, and reliability—dimensions that align closely with the core dimensions of trust (benevolence, competence, and integrity). Thus, while credibility often functions as a message‐level judgment and trust as a relational expectation over time, their theoretical foundations and communicative implications remain deeply interconnected.

For instance, Besley et al. (2012) defined credibility as the extent to which institutions are perceived as trustworthy, accurate, complete, and unbiased, positioning it as a predictor of public engagement behaviors such as attending public meetings. Bigham et al. (2019) further operationalized credibility as a combination of trustworthiness and expertise, showing that communicators perceived as trustworthy were rated as more credible and persuasive when discussing complex scientific topics. In a similar approach, Lederman et al. (2014) and Trumbo and McComas (2003) approached credibility through evaluative criteria such as fairness, accuracy, and verification, demonstrating that individuals rely on these cues to determine message reliability and to shape their processing of risk information. Across these studies, credibility is often framed as the cognitive foundation of trust, representing the initial judgments through which trust develops in scientific or risk communication contexts.

Including these works thus strengthens the theoretical base of the current review by situating trust within the credibility–trust continuum, where both constructs emphasize perceived integrity and expertise but differ in relational depth. While credibility‐based studies help explain how audiences assess communicative reliability, this review's focus on trust extends beyond message quality to examine how these perceptions evolve into ongoing expectations of institutional and interpersonal reliability. Therefore, credibility‐centered models inform—but are conceptually distinct from—the trust frameworks that underlie this study's analytical focus.

Furthermore, qualitative research examining the development of trust adds nuance to this discussion. Sillence et al. (2004) found through interviews and analysis that trust can be shown to unfold through a sequence of stages, including an initial rapid screening based on visual design heuristics, followed by a more deliberate evaluation of content credibility. Trust judgments evolve as participants gain experience with information; finding that trust maintenance depends on personalization and alignment with stakeholder values. Adding to this notion, Hendriks et al. (2016) demonstrated through qualitative interviews that when laypeople are asked open‐endedly to evaluate scientific experts, they spontaneously assess dimensions such as expertise, objectivity, work ethic, and potential conflicts of interest, further enhancing our understanding of the process of the development of trust.

Beyond functioning as a mediator or moderator, research has conceptualized trust as a dynamic phenomenon that unfolds procedurally across contexts and interactions. For instance, Agodi et al. (2024) show that credibility is not a set property of information but is negotiated through a social process in which individuals evaluate information according to practical needs and social interaction. Participants who expressed generalized trust in science granted legitimacy to claims rejected by scientific consensus when those claims aligned with trusted networks of peers or practical lived experiences. As the authors conclude, “socio‐pragmatic concerns make people shift back and forward in ascribing credibility to claims sustained in one epistemic community or another” (784), suggesting that trust and credibility are situated achievements rather than fixed attributes of either trustors or trustees.

### 4.7 Communicating Complex and Uncertain Information

Building on credibility‐based approaches, scholarship in science and risk communication applies theoretical frameworks that position trust at the center of how the public engages with complex or uncertain information. Drawing on perspectives such as the social amplification of risk framework (SARF), risk information seeking and processing (RISP) model, and risk communication theories (Renn 2010), these studies position communication as a process through which trust in science and institutions is constructed, maintained, or eroded.

Several studies have examined how scientists and institutions convey trustworthiness through message framing and relational engagement. For instance, Choi et al. (2023) found that scientists' willingness to correct misinformation was shaped by their attitudes toward the public: those adopting a dialogical orientation were more likely to engage relationally, while those adhering to a deficit model were less likely to intervene. Similarly, Markowitz (2024) illustrated that simplified AI‐generated scientific summaries enhanced perceived credibility and comprehension, although sometimes at the cost of perceived expertise—revealing an inherent tension between clarity and authority in communicating science.

Studies in environmental and technological risk contexts (e.g., Nakayachi and Ozaki 2014; Ryu et al. 2018; Seebauer and Babcicky 2018) consistently highlight that trust in institutions and local actors determines whether audiences interpret messages as credible and act upon them. Ryu et al. (2018) found that trust in regulatory bodies was a stronger predictor of nuclear policy acceptance than trust in government, while Nakayachi and Ozaki (2014) showed that shared fate—when risk managers personally experience the same risks as the public—significantly increases perceived trustworthiness. Wilkins et al. (2018) and Myrick and Hendryx (2021) extended these findings to environmental communication, showing that trust in scientific and interpersonal sources, rather than government or industry, drives the engagement and acceptance of environmental information.

Building on insights from these studies, several recommendations emerge for communicating complex or uncertain scientific knowledge without eroding trust. Effective communication requires transparency and empathy—acknowledging uncertainty and addressing public concerns directly rather than withholding information (Wilson et al. 2017; Yi et al. 2013). Trust is strengthened when experts demonstrate both competence and care, combining factual accuracy with an awareness of shared values and social implications (Nakayachi and Ozaki 2014; Ryu et al. 2018). Moreover, dialog‐based and participatory approaches—where publics are treated as partners, not passive recipients—foster legitimacy and reduce polarization (Choi et al. 2023; Wilkins et al. 2018). Finally, communicators should aim for clarity without oversimplification, ensuring accessibility while preserving scientific integrity (Markowitz 2024). Collectively, these practices reaffirm that sustaining trust in science depends less on authority and more on the relational ethics of openness, responsiveness, and respect, including perceptions of the communicating individual or organization.

## 5 Discussion

Understanding why people trust certain sources of information is critical in the context of misinformation, as trust often determines whether individuals accept or reject information, regardless of its accuracy. Trust, as defined by Hancock et al. (2023) and van Antwerpen et al. (2025), represents an individual's willingness to be vulnerable based on expectations of another's reliability and integrity. Within the dynamics of misinformation, stakeholder trust is multifaceted, shaped by the characteristics of the trustor, the trustee, and the context in which information is exchanged. These dynamics reveal that stakeholder trust operates simultaneously as a psychological process, a relational construct, and a communicative function.

### 5.1 Trust, Knowledge, and Misinformation

Recent research emphasizes misinformation within a broader social moment in which knowing and believing are not merely cognitive acts but also social (Kirmayer 2024). Public understanding of science depends on participation in epistemic communities—networks of experts, media, and citizens—that shape collective reasoning and determine what counts as credible knowledge. This framing underscores that efforts to address misinformation cannot be confined to correcting individual misconceptions; they must also strengthen the social and institutional infrastructures that sustain trustworthy communication. As Ognyanova et al. (2020) demonstrate, exposure to misinformation erodes confidence in traditional media but can increase stakeholder trust in partisan political institutions, especially when they reinforce existing worldviews. For researchers, this implies a need to examine trust as a context‐dependent social orientation—shaped by identity, ideology, or institutional performance.

The qualitative literature reviewed here reinforces this view by showing that trust is not a stable individual attribute but is actively constructed through webs of interaction and communication. Across multiple studies, trust has been shown to shift from vertical, institution‐centered frameworks toward horizontal peer networks, with social media platforms simultaneously enabling authorities to reach wider audiences and empowering alternative credibility communities that can either support or undermine scientific consensus (Stasik and Jemielniak 2022). The quality, consistency, and perceived legitimacy of official communications shape whether trust functions as a foundation for public compliance or erodes into skepticism, particularly as networked environments amplify both authoritative messages and misinformation (Shahbazi and Bunker 2024; Stasik and Jemielniak 2022).

As such, these findings suggest that stakeholder trust cannot be adequately captured at the level of the individual but must be studied as it circulates across actors, platforms, and institutions. Rather, the work—especially in the qualitative domain—suggests that there are networked and distributed aspects to trust that need to be further explored, leading to the following recommendation.

*Recommendation*: Future research should explore trust as a distributed social property rather than solely an individual trait, using network‐based and epistemic community frameworks to trace how credibility circulates through digital and institutional environments.

### 5.2 Trust and the Diffusion of Information

Studies examining the diffusion of misinformation show that trust has a critical role in how information and misinformation spread online. González‐Bailón et al. (2024) found that misinformation spreads more slowly than accurate news but also relies on small clusters of highly active users who act as credibility amplifiers within their networks. Similarly, Buchanan and Benson (2019) demonstrate that messages originating from trusted sources—regardless of accuracy—are more likely to be shared. Trust is therefore a transmission vector that enables the propagation of misinformation through relational endorsement rather than informational accuracy.

In sum, this underscores the importance of delineating between informational trust and relational trust. While much current research models diffusion through algorithmic or behavioral variables, relatively little addresses how perceived source trustworthiness mediates message spread.

*Recommendation*: Researchers should integrate trust measurement into models of information diffusion and examine how interpersonal and stakeholder trust interact with algorithmic visibility to shape virality in information environments.

### 5.3 Science Literacy, Epistemic Competence, and Corrective Interventions

Research on combating misinformation has increasingly emphasized the need for multifaceted strategies that combine science literacy education, technological interventions, and tailored corrective messaging. Sharon and Baram‐Tsabari (2020) and Allchin (2023) argue that literacy and education should go beyond factual understanding and encompass epistemic virtues such as open‐mindedness, intellectual humility, and intellectual courage. These dispositions reframe individuals to function as “competent outsiders”—nonexperts who can effectively assess scientific claims and engage with expert communities without needing to become experts themselves.

Central to this approach is the development of *epistemic dependence*, or the recognition that laypersons must often rely on scientific authorities while also acquiring the skills to distinguish legitimate expertise from deceptive or untrustworthy sources (Wang et al. 2022; Lederman et al. 2014; Peters et al. 1997). These include recognizing authentic expertise, identifying credible gatekeepers, detecting manipulation techniques (Erisen and Erisen 2025; Zhang et al. 2025), and fostering self‐regulation to mitigate cognitive biases such as confirmation bias (Small et al. 2014). These competencies are conceptualized across three levels: *epistemic motivation* (a commitment to trustworthy knowledge), *media reasoning* (understanding how scientific information is constructed and conveyed), and *personal interpretation* (awareness of one's cognitive limitations and vulnerabilities). This echoes work in information literacy, where scholars have long pointed to the importance of better understanding how consumers of information process and read, noting that even experts fall prey to official‐looking logos and domain names (i.e., Wineburg and McGrew 2019).

Di Sotto and Viviani (2022) extend this approach and show that automated systems—capable of analyzing textual, linguistic, network, and user‐profile features—can assist both experts and nonexperts in identifying health‐related misinformation across digital environments. These systems help manage the volume and complexity of online content, offering scalable solutions that can flag or reduce the spread of harmful information. However, while automated detection supports early identification, it does not address how users respond to corrective content. In this regard, Jennings and Stroud (2023) show that corrective interventions on social media are often filtered through motivated reasoning, where partisans interpret fact checks according to preexisting political identity. Ultimately, this leads us to the following recommendation regarding future approaches to developing a deeper understanding of trust, literacy, and media reasoning based on the current review.

*Recommendation*: Future research should integrate models of epistemic competence that combine science literacy, trust in institutions, and media reasoning skills. Experimental designs could, for instance, test information accuracy as well as credibility perceptions in polarized environments.

### 5.4 Consequences of Misinformation and the Impact of Trust

The body of reviewed literature demonstrates that the consequences of misinformation related to trust are particularly acute when the subject matter involves complex or uncertain knowledge domains, such as public health, climate science, or emerging technologies. In these contexts, the inherent ambiguity and technical difficulty of the subject matter make individuals more susceptible to simplified or misleading narratives (Ognyanova et al. 2020; Porter and Wood 2021). Social media accelerates the diffusion of such misinformation, as platforms reward emotionally resonant and easily digestible content—qualities often at odds with the nuance required to accurately communicate complex scientific issues (González‐Bailón et al. 2024). Motivated reasoning further compounds the problem; when individuals encounter information that aligns with their beliefs or ideological identities, they are more likely to accept it uncritically, even if it distorts or oversimplifies scientific facts (Jennings and Stroud 2023).

This tension between complexity and communicability poses challenges for interventions such as fact‐checking and content moderation. Fact checks often fail to correct misperceptions in politically polarized environments, especially when they involve highly technical, uncertain, or evolving knowledge—such as vaccine efficacy or climate models—that may be difficult for lay audiences to interpret. Moreover, even well‐intentioned moderation efforts struggle to contain misinformation that spreads through private channels, where peer‐to‐peer trust can override institutional authority (González‐Bailón et al. 2024). From a qualitative perspective, Bier (2001, 146) notes that much of the research on stakeholder participation and trust “has been qualitative and/or anecdotal” due to the richness of stakeholder interactions. In fact, qualitative research on stakeholder trust in this context has often aimed to capture the temporal unfolding of trust, the socio‐pragmatic forces that shape credibility, and participatory processes through which trust is built or eroded.

All these challenges point to the need to better understand the ramifications of misinformation spread over time on trust and, conversely, the impact of measures to counter misinformation over time (see Lewandowsky et al. 2012 for a deeper examination of countering misinformation). In this context and given the relative dearth of articles taking a longitudinal approach (*k* = 1 quantitative; *k* = 2 qualitative), there is a need for further longitudinal study in this domain.

*Recommendation*: Scholars should focus on longitudinal studies that measure how misinformation affects beliefs and actions, as well as institutional legitimacy over time. Moving beyond single snapshots in time is important for understanding the overriding impact of misinformation.

### 5.5 Toward a Relational Framework for Stakeholder Trust and Risk Communication

Taken together, the literature demonstrates that stakeholder trust is not purely cognitive or purely affective. There are critical studies focused on aspects that are relational, dynamic, and deeply contextual. Stakeholder trust emerges from reciprocal displays of credibility between communicators and audiences and is shaped by transparency, empathy, and shared values. In risk communication, the challenge is not simply to deliver accurate information but to cultivate durable trust networks capable of withstanding uncertainty and contestation. To advance this field, researchers should look to adopt multilevel and interdisciplinary designs that integrate communicative, sociological, and computational approaches such as large language models and the integration of artificial intelligence into the research workflow. Future research should focus on trust as an interactive system based on cocreation among institutions, intermediaries, and public stakeholders, rather than focusing on trust as an individual attitude or feature.

## 6 Conclusion

Addressing misinformation requires more than message accuracy. Countering misinformation demands engaging deeply with the communicative effects of trust in an age of information abundance. Furthermore, sustaining trust over time depends on transparency, accountability, and responsiveness, not on authority alone. As digital and scientific communication converges in online spaces, scholars must continue to examine how stakeholder trust can be built, maintained, and empirically measured across increasingly fragmented and algorithmically mediated spaces.

## Funding

This article is based on work supported by the U.S. Department of Energy, under Cooperative Agreement Number DE‐EM0005321 entitled ‘Consortium for Risk Evaluation with Stakeholder Participation (CRESP)’ awarded to Vanderbilt University. The opinions, findings, conclusions, or recommendations expressed herein are those of the authors and do not necessarily represent the views of the Department of Energy or Vanderbilt University.

## Supporting information




**Supporting Information**: risa70337‐supp‐0001‐SuppMat.docx

## References

[risa70337-bib-0001] Agodi, M. C. , I. Picardi , and L. Serafini . 2024. “The Socio‐Pragmatic Dimension of Truth. How Knowledge Claims Refused by Science Find Support in Public Discourse.” Rassegna Italiana di Sociologia, Rivista trimestrale fondata da Camillo Pellizzi LXIV, no. 4: 763–790.

[risa70337-bib-0002] Alabri, A. 2025. “Beyond Scanning and Seeking: The Role of Information Consumption Typologies in Shaping COVID‐19 Knowledge, Misinformation, Trust, and Vaccination Intentions.” Health Communication 40, no. 14: 3114–3125. 10.1080/10410236.2025.2497927. 40302607

[risa70337-bib-0003] Allchin, D. 2023. “Ten Competencies for the Science Misinformation Crisis.” Science Education 107, no. 2: 261–274. 10.1002/sce.21746.

[risa70337-bib-0004] Aven, T. , and S. A. Thekdi . 2022. “On How to Characterize and Confront Misinformation in a Risk Context.” Journal of Risk Research 25, no. 11–12: 1272–1287.

[risa70337-bib-0005] Balog‐Way, D. , and K. McComas . 2025. “Unpacking the Risk of Misinformation: A Communication‐Based Critique.” Risk Analysis 45, no. 12: 4097–4109. 10.1111/risa.70093. 40819939 PMC12747692

[risa70337-bib-0006] Barker, K. , E. Bessarabova , S. Radhakrishnan , et al. 2025. “Risk Analysis of Disinformation Weaponized Against Critical Networks.” Risk Analysis 45, no. 12: 4088–4096. 10.1111/risa.70062. 40623708

[risa70337-bib-0007] Besley, J. C. , K. A. McComas , and C. W. Trumbo . 2012. “Citizen Views About Public Meetings.” Journal of Risk Research 15, no. 4: 355–371. 10.1080/13669877.2011.634516.

[risa70337-bib-0008] Bier, V. 2001. “On the State of the Art: Risk Communication to the Public.” Reliability Engineering & System Safety 71, no. 2: 139–150. 10.1016/s0951-8320(00)00090-9.

[risa70337-bib-0009] Bigham, A. , C. Meyers , N. Li , and E. Irlbeck . 2019. “The Effect of Emphasizing Credibility Elements and the Role of Source Gender on Perceptions of Source Credibility.” Journal of Applied Communications 103, no. 2: 3. 10.4148/1051-0834.2270.

[risa70337-bib-0010] Buchanan, T. , and V. Benson . 2019. “Spreading Disinformation on Facebook: Do Trust in Message Source, Risk Propensity, or Personality Affect the Organic Reach of “Fake News”?” Social Media + Society 5, no. 4: 2056305119888654. 10.1177/2056305119888654.

[risa70337-bib-0011] Caled, D. , and M. J. Silva . 2022. “Digital Media and Misinformation: An Outlook on Multidisciplinary Strategies Against Manipulation.” Journal of Computational Social Science 5, no. 1: 123–159. 10.1007/s42001-021-00118-8. 34075349 PMC8156576

[risa70337-bib-0012] Choi, S. , A. A. Anderson , S. Cagle , M. Long , and N. Kelp . 2023. “Scientists' Deficit Perception of the Public Impedes Their Behavioral Intentions to Correct Misinformation.” PLoS One 18, no. 8: e0287870. 10.1371/journal.pone.0287870. 37531388 PMC10395896

[risa70337-bib-0013] Conchie, S. M. , and C. Burns . 2009. “Improving Occupational Safety: Using a Trusted Information Source to Communicate About Risk.” Journal of Risk Research 12, no. 1: 13–25. 10.1080/13669870802433749.

[risa70337-bib-0014] Corritore, C. L. , S. Wiedenbeck , B. Kracher , and R. P. Marble . 2012. “Online Trust and Health Information Websites.” International Journal of Technology and Human Interaction 8, no. 4: 92–115. 10.4018/jthi.2012100106.

[risa70337-bib-0015] Cvetkovich, G. , and K. Nakayachi . 2007. “Trust in a High‐Concern Risk Controversy: A Comparison of Three Concepts.” Journal of Risk Research 10, no. 2: 223–237. 10.1080/13669870601122519.

[risa70337-bib-0016] Di Sotto, S. , and M. Viviani . 2022. “Health Misinformation Detection in the Social Web: An Overview and a Data Science Approach.” International Journal of Environmental Research and Public Health 19, no. 4: 2173. 10.3390/ijerph19042173. 35206359 PMC8872515

[risa70337-bib-0017] Elisabetta Galeotti, A. , and C. Meini . 2022. “Scientific Misinformation and Fake News: A Blurred Boundary.” Social Epistemology 36, no. 6: 703–718.

[risa70337-bib-0018] Erisen, C. , and E. Erisen . 2025. “Populist Attitudes and Misinformation Challenging Trust: The Case of Turkey.” International Journal of Public Opinion Research 37, no. 1: edae056. 10.1093/ijpor/edae056.

[risa70337-bib-0019] Frewer, L. J. , J. Scholderer , and L. Bredahl . 2003. “Communicating About the Risks and Benefits of Genetically Modified Foods: The Mediating Role of Trust.” Risk Analysis 23, no. 6: 1117–1133. 10.1111/j.0272-4332.2003.00385.x. 14641888

[risa70337-bib-0020] Fujita, T. 2025. “Energy Knowledge and Public Response to Restarting Nuclear Plants in Japan Following the Fukushima Accident.” Utilities Policy 92: 101858. 10.1016/j.jup.2024.101858.

[risa70337-bib-0021] Gilmour, J. , R. Beilin , and T. Sysak . 2011. “Biosecurity Risk and Peri‐Urban Landholders—Using a Stakeholder Consultative Approach to Build a Risk Communication Strategy.” Journal of Risk Research 14, no. 3: 281–295. 10.1080/13669877.2010.528560.

[risa70337-bib-0022] González‐Bailón, S. , D. Lazer , P. Barberá , et al. 2024. “The Diffusion and Reach of (Mis)Information on Facebook During the U.S. 2020 Election.” Sociological Science 11: 1124–1146. 10.15195/v11.a41.

[risa70337-bib-0023] Guo, W. , and Q. Bai . 2023. “Pandemic and the Media: How Risk Information Seeking Influences Media Trust and Emotions.” Computers in Human Behavior 139: 107522. 10.1016/j.chb.2022.107522.

[risa70337-bib-0024] Hameleers, M. 2023. “Disinformation as a Context‐Bound Phenomenon: Toward a Conceptual Clarification Integrating Actors, Intentions and Techniques of Creation and Dissemination.” Communication Theory 33, no. 1: 1–10. 10.1093/ct/qtac021.

[risa70337-bib-0025] Hancock, P. A. , T. T. Kessler , A. D. Kaplan , et al. 2023. “How and Why Humans Trust: A Meta‐Analysis and Elaborated Model.” Frontiers in Psychology 14: 1081086. 10.3389/fpsyg.2023.1081086. 37051611 PMC10083508

[risa70337-bib-0026] Hendriks, F. , D. Kienhues , and R. Bromme . 2016. “Trust in Science and the Science of Trust.” In Trust and Communication in a Digitized World: Models and Concepts of Trust Research, edited by B. Blöbaum , 143–159. Springer International Publishing. 10.1007/978-3-319-28059-2_8.

[risa70337-bib-0027] Jennings, J. , and N. J. Stroud . 2023. “Asymmetric Adjustment: Partisanship and Correcting Misinformation on Facebook.” New Media & Society 25, no. 7: 1501–1521. 10.1177/14614448211021720.

[risa70337-bib-0028] Jin, X.‐L. , M. Yin , Z. Zhou , and X. Yu . 2021. “The Differential Effects of Trusting Beliefs on Social Media Users' Willingness to Adopt and Share Health Knowledge.” Information Processing & Management 58, no. 1: 102413. 10.1016/j.ipm.2020.102413.

[risa70337-bib-0029] Katsuya, T. 2002. “Difference in the Formation of Attitude Toward Nuclear Power.” Political Psychology 23, no. 1: 191–203. 10.1111/0162-895X.00277.

[risa70337-bib-0030] Kirmayer, L. J. 2024. “ *Science and Sanity*: A Social Epistemology of Misinformation, Disinformation, and the Limits of Knowledge.” Transcultural Psychiatry 61, no. 5: 795–808. 10.1177/13634615241296301. 39587900 PMC11629592

[risa70337-bib-0031] Kožuh, I. , and P. Čakš . 2021. “Explaining News Trust in Social Media News During the COVID‐19 Pandemic—The Role of a Need for Cognition and News Engagement.” International Journal of Environmental Research and Public Health 18, no. 24: 12986. 10.3390/ijerph182412986. 34948596 PMC8701362

[risa70337-bib-0032] Latkin, C. A. , L. Dayton , J. R. Miller , et al. 2021. “Behavioral and Attitudinal Correlates of Trusted Sources of COVID‐19 Vaccine Information in the US.” Behavioral Sciences 11, no. 4: 56. 10.3390/bs11040056. 33924118 PMC8074305

[risa70337-bib-0033] Lederman, R. , H. Fan , S. Smith , and S. Chang . 2014. “Who Can You Trust? Credibility Assessment in Online Health Forums.” Health Policy and Technology 3, no. 1: 13–25. 10.1016/j.hlpt.2013.11.003.

[risa70337-bib-0034] Lee, S. , and J. S. Jeong . 2024. “Information Environments Surrounding General Social Trust in COVID‐19 Context: A Model of Belief in Misinformation and In‐Group Trust.” International Journal of Communication 18: 24.

[risa70337-bib-0035] Lewandowsky, S. , U. K. H. Ecker , C. M. Seifert , N. Schwarz , and J. Cook . 2012. “Misinformation and Its Correction: Continued Influence and Successful Debiasing: Continued Influence and Successful Debiasing.” Psychological Science in the Public Interest 13, no. 3: 106–131. 26173286 10.1177/1529100612451018

[risa70337-bib-0036] Li, D. , &, and E. Santos Jr . 2020. “Discriminating Deception From Truth and Misinformation: An Intent‐Level Approach.” Journal of Experimental & Theoretical Artificial Intelligence 32, no. 3: 373–407. 10.1080/0952813X.2019.1652354.

[risa70337-bib-0037] Malka, A. , J. A. Krosnick , and G. Langer . 2009. “The Association of Knowledge With Concern About Global Warming: Trusted Information Sources Shape Public Thinking.” Risk Analysis 29, no. 5: 633–647. 10.1111/j.1539-6924.2009.01220.x. 19302280

[risa70337-bib-0038] Markowitz, D. M. 2024. “From Complexity to Clarity: How AI Enhances Perceptions of Scientists and the Public's Understanding of Science.” PNAS Nexus 3, no. 9: pgae387. 10.1093/pnasnexus/pgae387. 39290437 PMC11406778

[risa70337-bib-0039] Myrick, J. G. , and M. Hendryx . 2021. “Health Information Source Use and Trust Among a Vulnerable Rural Disparities Population.” Journal of Rural Health 37, no. 3: 537–544. 10.1111/jrh.12561. 33666269

[risa70337-bib-0040] Nakayachi, K. , and T. Ozaki . 2014. “A Method to Improve Trust in Disaster Risk Managers: Voluntary Action to Share a Common Fate.” International Journal of Disaster Risk Reduction 10: 59–66. 10.1016/j.ijdrr.2014.07.003.

[risa70337-bib-0041] Ndlela, M. N. 2018. “A Stakeholder Approach to Risk Management.” In Crisis Communication: A Stakeholder Approach, 53–75. Springer International Publishing. 10.1007/978-3-319-97256-5_4.

[risa70337-bib-0042] Ognyanova, K. , D. Lazer , R. E. Robertson , and C. Wilson . 2020. “Misinformation in Action: Fake News Exposure Is Linked to Lower Trust in Media, Higher Trust in Government When Your Side Is in Power.” Harvard Kennedy School Misinformation Review, June 2. 10.37016/mr-2020-024.

[risa70337-bib-0043] O'Neill, O. 2018. “Linking Trust to Trustworthiness.” International Journal of Philosophical Studies 26, no. 2: 293–300.

[risa70337-bib-0044] Page, M. J. , J. E. McKenzie , P. M. Bossuyt , et al. 2021. “The PRISMA 2020 Statement: An Updated Guideline for Reporting Systematic Reviews.” BMJ 372: n71. 33782057 10.1136/bmj.n71 PMC8005924

[risa70337-bib-0045] Peters, R. G. , V. T. Covello , and D. B. McCallum . 1997. “The Determinants of Trust and Credibility in Environmental Risk Communication: An Empirical Study.” Risk Analysis 17, no. 1: 43–54. 10.1111/j.1539-6924.1997.tb00842.x. 9131825

[risa70337-bib-0046] Peterson, E. B. , W.‐Y. S. Chou , D. E. Kelley , and B. Hesse . 2020. “Trust in National Health Information Sources in the United States: Comparing Predictors and Levels of Trust Across Three Health Domains.” Translational Behavioral Medicine 10, no. 4: 978–988. 10.1093/tbm/ibz066. 31116400 PMC7753001

[risa70337-bib-0047] Phogat, P. , S. Rab , and M. Wan . 2025. “Science Communication in the Digital Age: Trends, Gaps, and Interdisciplinary Opportunities.” Information Services and Use 45, no. 1–2: 148–163. 10.1177/18758789251342896.

[risa70337-bib-0048] Porter, E. , and T. J. Wood . 2021. “The Global Effectiveness of Fact‐Checking: Evidence From Simultaneous Experiments in Argentina, Nigeria, South Africa, and the United Kingdom.” Proceedings of the National Academy of Sciences of the United States of America 118, no. 37: e2104235118. 10.1073/pnas.2104235118. 34507996 PMC8449384

[risa70337-bib-0049] Renn, O. 2010. “Risk Communication: Insights and Requirements for Designing Successful Communication Programs on Health and Environmental Hazards.” In Handbook of Risk and Crisis Communication, edited by R. L. Health and H. D. O'Hair , 80–98. Routledge.

[risa70337-bib-0050] Renn, O. , and D. Levine . 1991. “Credibility and Trust in Risk Communication.” In Communicating Risks to the Public. Technology, Risk, and Society, edited by R.E. Kasperson and P. J. M. Stallen , 175–217. Springer. 10.1007/978-94-009-1952-5_10.

[risa70337-bib-0051] Rousseau, D. M. , S. B. Sitkin , R. S. Burt , and C. Camerer . 1998. “Not So Different After All: A Cross‐Discipline View of Trust.” Academy of Management Review 23, no. 3: 393–404. 10.5465/amr.1998.926617.

[risa70337-bib-0052] Rubin, G. J. , A. K. Chowdhury , and R. Amlôt . 2012. “How to Communicate With the Public About Chemical, Biological, Radiological, or Nuclear Terrorism: A Systematic Review of the Literature.” Biosecurity and Bioterrorism: Biodefense Strategy, Practice, and Science 10, no. 4: 383–395. 10.1089/bsp.2012.0043. 23216210

[risa70337-bib-0053] Ryu, Y. , S. Kim , and S. Kim . 2018. “Does Trust Matter? Analyzing the Impact of Trust on the Perceived Risk and Acceptance of Nuclear Power Energy.” Sustainability 10, no. 3: 758. 10.3390/su10030758.

[risa70337-bib-0054] Schuster, C. , and A. M. Scheu . 2026. “How Communication of Scientific Uncertainty Affects Trust in Science—A Systematic Review.” Risk Analysis 46, no. 5: e70233. 10.1111/risa.70233. 42014938

[risa70337-bib-0055] Seebauer, S. , and P. Babcicky . 2018. “Trust and the Communication of Flood Risks: Comparing the Roles of Local Governments, Volunteers in Emergency Services, and Neighbours.” Journal of Flood Risk Management 11, no. 3: 305–316. 10.1111/jfr3.12313. 32030096 PMC6991925

[risa70337-bib-0056] Shahbazi, M. , and D. Bunker . 2024. “Social Media Trust: Fighting Misinformation in the Time of Crisis.” International Journal of Information Management 77: 102780. 10.1016/j.ijinfomgt.2024.102780.

[risa70337-bib-0057] Shamayleh, G. , and Z. Arsel . 2026. “Digital Affective Encounters: The Relational Role of Content Circulation on Social Media.” Journal of Consumer Research 52, no. 5: 914–934. 10.1093/jcr/ucaf023.

[risa70337-bib-0058] Sharon, A. J. , and A. Baram‐Tsabari . 2020. “Can Science Literacy Help Individuals Identify Misinformation in Everyday Life?” Science Education 104, no. 5: 873–894. 10.1002/sce.21581.

[risa70337-bib-0059] Siegrist, M. , G. Cvetkovich , and C. Roth . 2000. “Salient Value Similarity, Social Trust, and Risk/Benefit Perception.” Risk Analysis 20, no. 3: 353–362. 10.1111/0272-4332.203034. 10949414

[risa70337-bib-0060] Sillence, E. , P. Briggs , L. Fishwick , and P. Harris . 2004. “Trust and Mistrust of Online Health Sites.” Paper presented at Proceedings of the SIGCHI Conference on Human Factors in Computing Systems, Vienna, Austria, April 24–29. 10.1145/985692.985776.

[risa70337-bib-0061] Small, M. J. , Ü. Güvenç , and M. L. DeKay . 2014. “When Can Scientific Studies Promote Consensus Among Conflicting Stakeholders?” Risk Analysis 34, no. 11: 1978–1994. 10.1111/risa.12237. 24954376

[risa70337-bib-0062] Song, M. , J. Elson , T. Nguyen , S. Obasi , J. Pintar , and D. Bastola . 2024. “Exploring Trust Dynamics in Health Information Systems: The Impact of Patients' Health Conditions on Information Source Preferences.” Frontiers in Public Health 12: 1478502. 10.3389/fpubh.2024.1478502. 39651474 PMC11622144

[risa70337-bib-0063] Stasik, A. , and D. Jemielniak . 2022. “Public Involvement in Risk Governance in the Internet Era: Impact of New Rules of Building Trust and Credibility.” Journal of Risk Research 25, no. 8: 991–1007. 10.1080/13669877.2020.1864008.

[risa70337-bib-0064] Swire‐Thompson, B. , and D. Lazer . 2020. “Public Health and Online Misinformation: Challenges and Recommendations.” Annual Review of Public Health 41, no. 1: 433–451. 10.1146/annurev-publhealth-040119-094127. 31874069

[risa70337-bib-0065] Ter Huurne, E. F. J. , and J. M. Gutteling . 2009. “How to Trust? The Importance of Self‐Efficacy and Social Trust in Public Responses to Industrial Risks.” Journal of Risk Research 12, no. 6: 809–824. 10.1080/13669870902726091.

[risa70337-bib-0066] Tetteh, E. K. , T. Combs , E. H. Geng , and V. R. McKay . 2022. “Public Health Information Seeking, Trust, and COVID‐19 Prevention Behaviors: Cross‐Sectional Study.” Journal of Medical Internet Research 24, no. 9: e37846. 10.2196/37846. 36084197 PMC9528929

[risa70337-bib-0067] Treem, J. W. 2012. “Communicating Expertise: Knowledge Performances in Professional‐Service Firms.” Communication Monographs 79, no. 1: 23–47.

[risa70337-bib-0068] Trumbo, C. W. , and K. A. McComas . 2003. “The Function of Credibility in Information Processing for Risk Perception.” Risk Analysis 23, no. 2: 343–353. 10.1111/1539-6924.00313. 12731818

[risa70337-bib-0069] Vainio, A. , R. Paloniemi , and V. Varho . 2017. “Weighing the Risks of Nuclear Energy and Climate Change: Trust in Different Information Sources, Perceived Risks, and Willingness to Pay for Alternatives to Nuclear Power.” Risk Analysis 37, no. 3: 557–569. 10.1111/risa.12640. 27350103

[risa70337-bib-0070] van Antwerpen, N. , E. B. Green , D. Sturman , and R. A. Searston . 2025. “The Impacts of Expertise, Conflict, and Scientific Literacy on Trust and Belief in Scientific Disagreements.” Scientific Reports 15, no. 1: 11869. 10.1038/s41598-025-96333-8. 40195428 PMC11977003

[risa70337-bib-0078] Wang, W. , L. Atkinson , L. A. Kahlor , P. Jamar , and H. S. Lim . 2022. “Avoiding Covid‐19 Risk Information in the United States: the Role of Attitudes, Norms, Affect, Social Dominance Orientations, and Perceived Trustworthiness of Scientists.” Risk Analysis 43, no. 6: 1145–1161. Portico. 10.1111/risa.13991. 35790468 PMC9349373

[risa70337-bib-0071] Wang, Y. , H. Hao , and L. S. Platt . 2021. “Examining Risk and Crisis Communications of Government Agencies and Stakeholders During Early‐Stages of COVID‐19 on Twitter.” Computers in Human Behavior 114: 106568. 10.1016/j.chb.2020.106568. 32982038 PMC7508681

[risa70337-bib-0072] White, M. P. , and B. B. Johnson . 2010. “The Intuitive Detection Theorist (IDT) Model of Trust in Hazard Managers: IDT Model of Trust in Hazard Managers.” Risk Analysis 30, no. 8: 1196–1209. 10.1111/j.1539-6924.2010.01407.x. 20409046

[risa70337-bib-0073] Wilkins, E. J. , H. M. Miller , E. Tilak , and R. M. Schuster . 2018. “Communicating Information on Nature‐Related Topics: Preferred Information Channels and Trust in Sources.” PLoS One 13, no. 12: e0209013. 10.1371/journal.pone.0209013. 30540834 PMC6291159

[risa70337-bib-0074] Wilson, M. J. , T. L. Ramey , M. R. Donaldson , R. R. Germain , and E. K. Perkin . 2017. “Communicating Science: Sending the Right Message to the Right Audience.” FACETS 1: 127–137. 10.1139/facets-2016-0015.

[risa70337-bib-0075] Wineburg, S. , and S. McGrew . 2019. “Lateral Reading and the Nature of Expertise: Reading Less and Learning More When Evaluating Digital Information.” Teachers College Record 121, no. 11: 1–40.32508373

[risa70337-bib-0076] Yi, M. Y. , J. J. Yoon , J. M. Davis , and T. Lee . 2013. “Untangling the Antecedents of Initial Trust in Web‐Based Health Information: The Roles of Argument Quality, Source Expertise, and User Perceptions of Information Quality and Risk.” Decision Support Systems 55, no. 1: 284–295. 10.1016/j.dss.2013.01.029.

[risa70337-bib-0077] Zhang, X. , N. Lou , and M. Li . 2025. “Perceived Risk and Trust in News Sources: A Multi‐Group Analysis of Deepfakes and AI‐Generated Misinformation.” Journal of Broadcasting & Electronic Media 69, no. 3: 219–253. 10.1080/08838151.2025.2487050.

