# Supplementary material for: Communicating Risk in the Age of Misinformation: Empirical Frameworks for Understanding Stakeholder Trust
Source: Risk Anal. 2026 Aug 21;46(9):e70337. doi: 10.1111/risa.70337 (PMC13498723; doi:10.1111/risa.70337)
Supplement: Supplementary file 1 [file RISA-46-0-s001.docx]

Supplementary materials

[APPENDIX 1: DETAILED CLARIFICATION OF INCLUSION/EXCLUSION CRITERIA	2](#_Toc150263809)

[APPENDIX 2: SEARCH QUERIES	5](#_Toc150263811)

[APPENDIX 3: ARTICLES INCLUDED IN THE REVIEW	6](#_Toc150263813)

[APPENDIX 4: JOURNAL TOPICS	11](#_Toc150263814)

[APPENDIX 5: EFFECT SIZES OF QUANTITATIVE ARTICLES IN THE CORPUS	13](#_Toc150263815)

**Appendix 1: Detailed clarification of inclusion/exclusion criteria**

**A1.1. Justification for inclusion/exclusion of articles based on trust frameworks**

The review examines how stakeholder trust is conceptualized, operationalized, and integrated into communication processes in risk-involved or scientifically complex contexts. A central challenge in the inclusion process was distinguishing between studies that explicitly grounded their analysis in trust frameworks and those that addressed related constructs—such as credibility, confidence, or institutional legitimacy—without fully theorizing trust as a relational or mediating construct. Consequently, three guiding criteria were developed to ensure conceptual consistency and methodological rigor.

First, articles were included when trust was theoretically or empirically central to explaining communication, perception, or behavioral outcomes related to risk or scientific uncertainty. These studies framed trust as a psychological, relational, or institutional mechanism mediating between information exposure and audience response. Examples include research using risk communication frameworks (e.g., Risk Information Seeking and Processing [RISP]; Social Amplification of Risk Framework [SARF]) or models that conceptualize trust in science, institutions, or communicators as a determinant of message acceptance and engagement. Articles in which trust was explicitly operationalized through validated scales or multidimensional constructs (competence, integrity, benevolence) were prioritized.

Second, articles were included when trust was explicitly operationalized and empirically measured rather than assumed or inferred. These included studies employing validated or context-specific trust measures reflecting competence, integrity, and benevolence—the most widely recognized dimensions of trust across organizational and risk contexts. Works that used self-report instruments capturing “trust in government,” “trust in scientists,” or “trust in regulatory agencies” were also included if such measures were theoretically linked to communication processes or decision-making under uncertainty.

Third, studies were excluded when trust appeared only as a background condition or contextual descriptor without theoretical elaboration or empirical testing. For example, papers that referenced trust merely as part of a general discussion of public attitudes or institutional performance were omitted.

These criteria ensured that the final corpus included studies in which trust functioned as a theoretically specified and empirically examined mechanism, rather than merely appearing as an undefined background condition.

**A1.2. Justification for inclusion/exclusion of articles based on credibility**

The inclusion and exclusion of articles addressing credibility were guided by the distinction between credibility as believability and trust as relational confidence. While often related, credibility and trust differ conceptually: credibility concerns perceptions of a source’s reliability and expertise, whereas trust reflects the willingness to rely on that source and accept its information as valid. Credibility thus represents a cognitive evaluation of message reliability, while trust entails a relational and affective dimension involving vulnerability and positive expectations of the communicator.

Following this distinction, the review included only studies in which credibility was operationalized through trust-related dimensions—such as trustworthiness, integrity, or benevolence—and excluded those that examined credibility purely as a message- or source-level attribute. For instance, Besley et al. (2011), Bigham et al. (2019), and Lederman et al. (2014) conceptualized credibility as a combination of trustworthiness and expertise, demonstrating how perceptions of honesty and competence foster relational trust in scientific and risk communication. These studies were retained because credibility was treated as an antecedent or operational component of trust.

Conversely, articles that examined source credibility as an independent persuasive factor—without linking it to relational trust—were excluded. For example, Li and Feng (2025) treated credibility as a moderating cue for information believability in the context of media exposure and risk perception, while Abunyewah et al. (2019) defined credibility narrowly through message reliability and expertise. Both studies conceptualized credibility in cognitive rather than relational terms and therefore fell outside the scope of trust-based frameworks.

Therefore, articles were included only when credibility contributed to explaining how audiences form or reinforce trust in communicators or institutions. This distinction ensured that the review remained theoretically consistent, focusing on frameworks where credibility functions as a foundation for trust, rather than as a standalone construct of source believability.

**A1.3. Justification for inclusion/exclusion of articles based on risk-involved context**

The inclusion criteria for this review emphasized studies conducted in risk-involved communication contexts, where uncertainty, potential harm, and institutional responsibility influence how information is communicated, interpreted, and acted upon. The review included articles that addressed environmental risk, public health, safety, and science communication, as these settings require the negotiation of trust under conditions of ambiguity and perceived vulnerability. Across these studies, trust was examined as an independent, dependent, or mediating variable, reflecting its multifaceted role in shaping how individuals assess information credibility, perceive institutional competence, and decide whether to cooperate with or challenge expert authority.

In contrast, articles situated in consumer, marketing, and branding contexts were excluded. While such studies often reference trust, reliability, or credibility, they typically conceptualize trust as transactional—focused on consumer loyalty, satisfaction, or brand reputation rather than communicative trust within socially consequential or risk-laden settings. Similarly, studies centered on technological contexts such as the Internet of Things (IoT), artificial intelligence, or blockchain systems were excluded when trust was defined as system performance or reliability rather than as a relational construct embedded in human or institutional communication. For instance, research examining trust in digital algorithms, autonomous systems, or decentralized technologies was deemed outside the scope, as these contexts emphasize functional or technical assurance rather than the communicative and social dimensions of trust.

Ultimately, included studies were those in which trust was examined in the management, perception, or communication of risk—for example, in contexts involving environmental hazards, occupational safety, health crises, or institutional transparency. This focus ensured the conceptual coherence of the corpus by centering on research that investigates trust as part of the broader social process of understanding and responding to uncertainty, rather than as a construct confined to consumer decision-making or technological adoption.

**Appendix 2: Search Queries**

| table: search queries by database | |
| --- | --- |
| Web of Science | TS=((("trust*" OR "organizational trust" OR "interpersonal trust" OR "institutional trust" OR "dimensions of trust" OR "trusted") AND ("credibility" OR "information credibility" OR "source credibility" OR "institutional credibility" OR "information source*" OR "misinformation") AND ("risk*" OR "risk communication" OR "risk management" OR "risk perception*" OR "perception of risk*" OR "science communication") AND ("framework" OR "model*"))) |
| Scopus | TITLE-ABS-KEY = (  (  ("trust*" OR "organizational trust" OR "interpersonal trust" OR "institutional trust" OR "dimensions of trust" OR "trusted")  AND  ("credibility" OR "information credibility" OR "source credibility" OR "institutional credibility" OR "information source*" OR "misinformation")  AND  ("risk*" OR "risk communication" OR "risk management" OR "risk perception*" OR "perception of risk*" OR "science communication")  AND  ("framework*" OR "model*" OR "conceptual framework" OR "theoretical model")  )  ) |

**Appendix 3: Articles included in the review**

1. Agodi, M. C., Picardi, I., & Serafini, L. (2023). The socio-pragmatic dimension of truth. How knowledge claims refused by science find support in public discourse. Rassegna Italiana di Sociologia, Rivista trimestrale fondata da Camillo Pellizzi, (4).
2. Alabri, A. (2025). Beyond scanning and seeking: The role of information consumption typologies in shaping COVID-19 knowledge, misinformation, trust, and vaccination intentions. Health Communication, 1-12. <https://doi.org/10.1080/10410236.2025.2497927>
3. Baxter, J. (2009). A quantitative assessment of the insider/outsider dimension of the cultural theory of risk and place. Journal of Risk Research, 12(6), 771-791. <https://doi.org/10.1080/13669870802579806>
4. Bearth, A., & Siegrist, M. (2021). The social amplification of risk framework: A normative perspective on trust? Risk Analysis, 42(7), 1381-1392. <https://doi.org/10.1111/risa.13757>
5. Besley, J. C., McComas, K. A., & Trumbo, C. W. (2011). Citizen views about public meetings. Journal of Risk Research, 15(4), 355-371. <https://doi.org/10.1080/13669877.2011.634516>
6. Bier, V. (2001). On the state of the art: Risk communication to the public. Reliability Engineering & System Safety, 71(2), 139-150. <https://doi.org/10.1016/s0951-8320(00)00090-9>
7. Bigham, A., Meyers, C., Li, N., & Irlbeck, E. (2019). The effect of emphasizing credibility elements and the role of source gender on perceptions of source credibility. Journal of Applied Communications, 103(2). <https://doi.org/10.4148/1051-0834.2270>
8. Blöbaum, B. (2016). Key factors in the process of trust. On the analysis of trust under digital conditions. Progress in IS, 3-25. <https://doi.org/10.1007/978-3-319-28059-2_1>
9. Breakwell, G. M., & Jaspal, R. (2020). Identity change, uncertainty and mistrust in relation to fear and risk of COVID-19. Journal of Risk Research, 24(3-4), 335-351. <https://doi.org/10.1080/13669877.2020.1864011>
10. Burger, J. (2022). Ecological information and approaches needed for risk communication dialogs for acute or chronic environmental crises. Risk Analysis, 42(11), 2408-2420. <https://doi.org/10.1111/risa.13940>
11. Cairns, G., De Andrade, M., & MacDonald, L. (2013). Reputation, relationships, risk communication, and the role of trust in the prevention and control of communicable disease: A review. Journal of Health Communication, 18(12), 1550-1565. <https://doi.org/10.1080/10810730.2013.840696>
12. Castelfranchi, C., Falcone, R., & Pezzulo, G. (2003). Trust in information sources as a source for trust. Proceedings of the second international joint conference on Autonomous agents and multiagent systems - AAMAS '03. <https://doi.org/10.1145/860586.860590>
13. Chawla, U., Mohnot, R., Singh, H. V., & Banerjee, A. (2023). The mediating effect of perceived trust in the adoption of cutting-edge financial technology among digital natives in the Post-COVID-19 era. Economies, 11(12), 286. <https://doi.org/10.3390/economies11120286>
14. Choi, S., Anderson, A. A., Cagle, S., Long, M., & Kelp, N. (2023). Scientists’ deficit perception of the public impedes their behavioral intentions to correct misinformation. PLOS ONE, 18(8), e0287870. <https://doi.org/10.1371/journal.pone.0287870>
15. Conchie, S. M., & Burns, C. (2009). Improving occupational safety: Using a trusted information source to communicate about risk. Journal of Risk Research, 12(1), 13-25. <https://doi.org/10.1080/13669870802433749>
16. Corritore, C. L., Wiedenbeck, S., Kracher, B., & Marble, R. P. (2012). Online trust and health information websites. International Journal of Technology and Human Interaction, 8(4), 92-115. <https://doi.org/10.4018/jthi.2012100106>
17. Cvetkovich, G., & Nakayachi, K. (2007). Trust in a high?concern risk controversy: A comparison of three concepts. Journal of Risk Research, 10(2), 223-237. <https://doi.org/10.1080/13669870601122519>
18. Erisen, C., & Erisen, E. (2025). Populist attitudes and misinformation challenging trust: The case of Turkey. International Journal of Public Opinion Research, 37(1). <https://doi.org/10.1093/ijpor/edae056>
19. Fiske, S. T., & Dupree, C. (2014). Gaining trust as well as respect in communicating to motivated audiences about science topics. Proceedings of the National Academy of Sciences, 111(supplement_4), 13593-13597. <https://doi.org/10.1073/pnas.1317505111>
20. Frewer, L. J., Scholderer, J., & Bredahl, L. (2003). Communicating about the risks and benefits of genetically modified foods: The mediating role of trust. Risk Analysis, 23(6), 1117-1133. <https://doi.org/10.1111/j.0272-4332.2003.00385.x>
21. Fujita, T. (2025). Energy knowledge and public response to restarting nuclear plants in Japan following the Fukushima accident. Utilities Policy, 92, 101858. <https://doi.org/10.1016/j.jup.2024.101858>
22. Giardini, F., & Vilone, D. (2021). Opinion dynamics and collective risk perception: An agent-based model of institutional and media communication about disasters. Journal of Artificial Societies and Social Simulation, 24(1). <https://doi.org/10.18564/jasss.4479>
23. Gilmour, J., Beilin, R., & Sysak, T. (2021). Africa sustainable livestock 2050: Women and biosecurity practices in urban and peri-urban poultry value chains. Journal of Risk Research, 14(3), 281-295. <https://doi.org/10.4060/cb8077en>
24. Goodwin, J., & Dahlstrom, M. F. (2013). Communication strategies for earning trust in climate change debates. WIREs Climate Change, 5(1), 151-160. <https://doi.org/10.1002/wcc.262>
25. Greenberg, M. R. (2014). Energy policy and research: The underappreciation of trust. Energy Research & Social Science, 1, 152-160. <https://doi.org/10.1016/j.erss.2014.02.004>
26. Grosser, K. M. (2016). Trust in online journalism. Digital Journalism, 4(8), 1036-1057. <https://doi.org/10.1080/21670811.2015.1127174>
27. Gugliermetti, F., & Guidi, G. (2009). Risk communication for industrial plants and radioactive waste repositories. In W. Martorell, C. Guedes Soares, & J. Barnett (Eds.), Safety, reliability and risk analysis: Theory, methods and applications (pp. 1341-1349). Taylor & Francis Group.
28. Guo, W., & Bai, Q. (2023). Pandemic and the media: How risk information seeking influences media trust and emotions. Computers in Human Behavior, 139, 107522. <https://doi.org/10.1016/j.chb.2022.107522>
29. Hendriks, F., Kienhues, D., & Bromme, R. (2016). Trust in science and the science of trust. In B. Blöbaum (Ed.), Trust and communication in a digitized world: Models and concepts of trust research (pp. 143-159). Springer International Publishing. <https://doi.org/10.1007/978-3-319-28059-2_8>
30. Jabeen, F., Hamid, Z., Akhunzada, A., Abdul, W., & Ghouzali, S. (2018). Trust and reputation management in healthcare systems: Taxonomy, requirements and open issues. IEEE Access, 6, 17246-17263. <https://doi.org/10.1109/access.2018.2810337>
31. Jin, X., Yin, M., Zhou, Z., & Yu, X. (2021). The differential effects of trusting beliefs on social media users’ willingness to adopt and share health knowledge. Information Processing & Management, 58(1), 102413. <https://doi.org/10.1016/j.ipm.2020.102413>
32. Katsuya, T. (2002). Difference in the formation of attitude toward nuclear power. Political Psychology, 23(1), 191-203. <https://doi.org/10.1111/0162-895x.00277>
33. Kozuh, I., & Čakš, P. (2021). Explaining news trust in social media news during the COVID-19 pandemic - the role of a need for cognition and news engagement. International Journal of Environmental Research and Public Health, 18(24), 12986. <https://doi.org/10.3390/ijerph182412986>
34. Latkin, C. A., Dayton, L., Miller, J. R., Yi, G., Jaleel, A., Nwosu, C. C., Yang, C., & Falade-Nwulia, O. (2021). Behavioral and attitudinal correlates of trusted sources of COVID-19 vaccine information in the US. Behavioral Sciences, 11(4), 56. <https://doi.org/10.3390/bs11040056>
35. Lederman, R., Fan, H., Smith, S., & Chang, S. (2014). Who can you trust? Credibility assessment in online health forums. Health Policy and Technology, 3(1), 13-25. <https://doi.org/10.1016/j.hlpt.2013.11.003>
36. Lee, S., & Jeong, J. S. (2024). Information Environments Surrounding General Social Trust in COVID-19 Context: A Model of Belief in Misinformation and In-Group Trust. International journal of communication [Online], 18, 4370+. <https://link-gale-com.proxy.libraries.rutgers.edu/apps/doc/A812519122/LitRC?u=new67449&sid=summon&xid=1b539c50>
37. Markowitz, D. M. (2024). From complexity to clarity: How AI enhances perceptions of scientists and the public's understanding of science. PNAS Nexus, 3(9). <https://doi.org/10.1093/pnasnexus/pgae387>
38. Mitchell, J. V. (1992). Perception of risk and credibility at toxic sites. Risk Analysis, 12(1), 19-26. <https://doi.org/10.1111/j.1539-6924.1992.tb01303.x>
39. Myrick, J. G., & Hendryx, M. (2021). Health information source use and trust among a vulnerable rural disparities population. The Journal of Rural Health, 37(3), 537-544. <https://doi.org/10.1111/jrh.12561>
40. Nakayachi, K., & Ozaki, T. (2014). A method to improve trust in disaster risk managers: Voluntary action to share a common fate. International Journal of Disaster Risk Reduction, 10, 59-66. <https://doi.org/10.1016/j.ijdrr.2014.07.003>
41. Patent, V. (2022). Dysfunctional trusting and distrusting: Integrating trust and bias perspectives. Journal of Trust Research, 12(1), 66-93. <https://doi.org/10.1080/21515581.2022.2113887>
42. Peters, R. G., Covello, V. T., & McCallum, D. B. (1997). The Determinants of Trust and Credibility in Environmental Risk Communication: An Empirical Study. *Risk analysis*, *17*(1), 43-54. <https://doi.org/10.1111/j.1539-6924.1997.tb00842.x>
43. Peterson, E. B., Chou, W. S., Kelley, D. E., & Hesse, B. (2020). Trust in national health information sources in the United States: Comparing predictors and levels of trust across three health domains. Translational Behavioral Medicine, 10(4), 978-988. <https://doi.org/10.1093/tbm/ibz066>
44. Poortinga, W., & Pidgeon, N. F. (2003). Exploring the dimensionality of trust in risk regulation. Risk Analysis, 23(5), 961-972. <https://doi.org/10.1111/1539-6924.00373>
45. Prades, A., Oltra, C., Navajas, J., Horlick-Jones, T., & Espluga, J. (2009). The social perception of nuclear fusion: Investigating lay understanding and reasoning about the technology. In W. Martorell, C. Guedes Soares, & J. Barnett (Eds.), Safety, reliability and risk analysis: Theory, methods and applications (pp. 1371-1374). Taylor & Francis Group.
46. Reynolds, B., & Quinn, S. C. (2008). Effective communication during an influenza pandemic: The value of using a crisis and emergency risk communication framework. Health Promotion Practice, 9(4_suppl), 13S-17S. <https://doi.org/10.1177/1524839908325267>
47. Rubin, G. J., Chowdhury, A. K., & Amlôt, R. (2012). How to communicate with the public about chemical, biological, radiological, or nuclear terrorism: A systematic review of the literature. Biosecurity and Bioterrorism: Biodefense Strategy, Practice, and Science, 10(4), 383-395. <https://doi.org/10.1089/bsp.2012.0043>
48. Ryu, Y., Kim, S., & Kim, S. (2018). Does trust matter? Analyzing the impact of trust on the perceived risk and acceptance of nuclear power energy. Sustainability, 10(3), 758. <https://doi.org/10.3390/su10030758>
49. Seebauer, S., & Babcicky, P. (2017). Trust and the communication of flood risks: Comparing the roles of local governments, volunteers in emergency services, and neighbours. Journal of Flood Risk Management, 11(3), 305-316. <https://doi.org/10.1111/jfr3.12313>
50. Shahbazi, M., & Bunker, D. (2024). Social media trust: Fighting misinformation in the time of crisis. International Journal of Information Management, 77, 102780. <https://doi.org/10.1016/j.ijinfomgt.2024.102780>
51. Siegrist, M., Cvetkovich, G., & Roth, C. (2000). Salient value similarity, social trust, and risk/enefit perception. Risk Analysis, 20(3), 353-362. <https://doi.org/10.1111/0272-4332.203034>
52. Sillence, E., Briggs, P., Fishwick, L., & Harris, P. (2004). Trust and mistrust of online health sites. Proceedings of the SIGCHI Conference on Human Factors in Computing Systems, 663-670. <https://doi.org/10.1145/985692.985776>
53. Small, M. J., Güvenç, Ü., & DeKay, M. L. (2014). When can scientific studies promote consensus among conflicting stakeholders? Risk Analysis, 34(11), 1978-1994. <https://doi.org/10.1111/risa.12237>
54. Song, M., Elson, J., Nguyen, T., Obasi, S., Pintar, J., & Bastola, D. (2024). Exploring trust dynamics in health information systems: The impact of patients health conditions on information source preferences. Frontiers in Public Health, 12. <https://doi.org/10.3389/fpubh.2024.1478502>
55. Stasik, A., & Jemielniak, D. (2021). Public involvement in risk governance in the internet era: Impact of new rules of building trust and credibility. Journal of Risk Research, 25(8), 991-1007. <https://doi.org/10.1080/13669877.2020.1864008>
56. Stern, M. J., & Coleman, K. J. (2019). Trust ecology and collaborative natural resource management. A New Era for Collaborative Forest Management, 45-58. <https://doi.org/10.4324/9781351033381-3>
57. Ter Huurne, E. F., & Gutteling, J. M. (2009). How to trust? The importance of self-efficacy and social trust in public responses to industrial risks. Journal of Risk Research, 12(6), 809-824. <https://doi.org/10.1080/13669870902726091>
58. Tetteh, E. K., Combs, T., Geng, E. H., & McKay, V. R. (2022). Public health information seeking, trust, and COVID-19 prevention behaviors: Cross-sectional study. Journal of Medical Internet Research, 24(9), e37846. <https://doi.org/10.2196/37846>
59. Trumbo, C. W., & McComas, K. A. (2003). The function of credibility in information processing for risk perception. Risk Analysis, 23(2), 343-353. <https://doi.org/10.1111/1539-6924.00313>
60. Vainio, A., Paloniemi, R., & Varho, V. (2016). Weighing the risks of nuclear energy and climate change: Trust in different information sources, perceived risks, and willingness to pay for alternatives to nuclear power. Risk Analysis, 37(3), 557-569. <https://doi.org/10.1111/risa.12640>
61. Vanacker, B., & Belmas, G. (2009). Trust and the economics of news. Journal of Mass Media Ethics, 24(2-3), 110-126. <https://doi.org/10.1080/08900520902885277>
62. Wang, W., Atkinson, L., Kahlor, L. A., Jamar, P., & Lim, H. S. (2022). Avoiding covid?19 risk information in the United States: The role of attitudes, norms, affect, social dominance orientations, and perceived trustworthiness of scientists. Risk Analysis, 43(6), 1145-1161. <https://doi.org/10.1111/risa.13991>
63. Weingart, P., & Guenther, L. (2016). Science communication and the issue of trust. Journal of Science Communication, 15(05), C01. <https://doi.org/10.22323/2.15050301>
64. White, M. P., & Johnson, B. B. (2010). The intuitive detection theorist (IDT) model of trust in hazard managers. Risk Analysis, 30(8), 1196-1209. <https://doi.org/10.1111/j.1539-6924.2010.01407.x>
65. Wilkins, E. J., Miller, H. M., Tilak, E., & Schuster, R. M. (2018). Communicating information on nature-related topics: Preferred information channels and trust in sources. PLOS ONE, 13(12), e0209013. <https://doi.org/10.1371/journal.pone.0209013>
66. Wilson, M. J., Ramey, T. L., Donaldson, M. R., Germain, R. R., & Perkin, E. K. (2016). Communicating science: Sending the right message to the right audience. FACETS, 1, 127-137. <https://doi.org/10.1139/facets-2016-0015>
67. Yi, M. Y., Yoon, J. J., Davis, J. M., & Lee, T. (2013). Untangling the antecedents of initial trust in web-based health information: The roles of argument quality, source expertise, and user perceptions of information quality and risk. Decision Support Systems, 55(1), 284-295. <https://doi.org/10.1016/j.dss.2013.01.029>
68. Zhang, X., Lou, N., & Li, M. (2025). Perceived risk and trust in news sources: A multi-group analysis of Deepfakes and AI-generated misinformation. Journal of Broadcasting & Electronic Media, 69(3), 219-253. <https://doi.org/10.1080/08838151.2025.2487050>
69. Zhu, W., Wei, J., & Zhao, D. (2016). Anti-nuclear behavioral intentions: The role of perceived knowledge, information processing, and risk perception. Energy Policy, 88, 168-177. <https://doi.org/10.1016/j.enpol.2015.10.009>

**Appendix 4: Journal topics**

| Journal Topics | |
| --- | --- |
| Topic | Journals |
| Risk, hazards, safety | Risk Analysis |
|  | Journal of Risk Research |
|  | Reliability Engineering & System Safety |
|  | Decision Support Systems |
|  | International Journal of Disaster Risk Reduction |
|  | Journal of Flood Risk Management |
|  | Utilities Policy |
|  | Biosecurity and Bioterrorism |
| Communication | Environmental Communication |
|  | Health Communication |
|  | Journal of Health Communication |
|  | Journal of Applied Communications |
|  | Journal of Science Communication |
| Environment and energy | Energy Research & Social Science |
|  | Sustainability |
|  | Energy Policy |
|  | Energy Research & Social Science |
| Health | Frontiers in Public Health |
|  | Health Policy and Technology |
|  | Translational Behavioral Medicine |
|  | The Journal of Rural Health |
|  | PEC Innovation |
| Information and technology | Computers in Human Behavior |
|  | Information Processing & Management |
|  | International Journal of Information Management |
|  | IEEE Access |
|  | Progress in IS |
|  | International Journal of Technology and Human Interaction |
|  | Journal of Artificial Societies and Social Simulation |
| Digital media | Social Media + Society |
|  | Digital Journalism |
|  | Journal of Broadcasting & Electronic Media |
| Sociology and psychology | Political Psychology |
|  | Journal of Trust Research |
|  | Behavioral Sciences |
|  | Economies |
|  | International Journal of Public Opinion Research |
| Interdisciplinary science | Public Understanding of Science |
|  | PNAS Nexus |
|  | FACETS |
|  | Decision Support Systems |

Appendix 5: Effect sizes of relevant quantitative articles in the corpus

| Article | Risk topic | Variables | Statistic | Valence | Reported statistical associations and effect estimates | Significance |
| --- | --- | --- | --- | --- | --- | --- |
| Alabri (2025) | COVID-19 | LSS → trust in government | β | Pos | 0.19 | ** |
|  |  | LSS → trust in social media |  | Pos | 0.12 | * |
|  |  | HSNS → trust in government |  | Pos | 0.09 | * |
|  |  | HSNS → trust in social media |  | Pos | 0.11 | * |
|  |  | HSS → trust in government |  | Pos | 0.35 | ** |
|  |  | HSS → trust in social media |  | Pos | 0.46 | ** |
|  |  | HSS → institutional trust |  | Pos | 0.15 | ** |
|  |  | Past hygiene adherence → trust in government |  | Pos | 0.24 | ** |
|  |  | Past hygiene adherence → institutional trust |  | Pos | 0.21 | ** |
|  |  | Health literacy → trust in government |  | Pos | 0.16 | ** |
|  |  | Health literacy → institutional trust |  | Pos | 0.15 | ** |
|  |  | Perceived susceptibility → trust in social media |  | Neg | -0.14 | ** |
|  |  | Perceived severity → trust in social media |  | Pos | 0.14 | ** |
|  |  | Trust in government → vaccination intentions |  | Pos | 0.11 | ** |
|  |  | Past hygiene adherence → vaccination intentions |  | Pos | 0.11 | * |
|  |  | Health literacy → vaccination intentions |  | Pos | 0.14 | ** |
|  |  | Institutional trust → vaccination intentions |  | Pos | 0.14 | ** |
| Baxter (2009) | Communities living w/ technological environmental hazards | Trust in outside media → facility-related concern (pooled) | OR | Pos | 1.77 | * |
|  |  | Trust in environmental groups → concern (pooled) |  | Pos | 3.06 | ** |
|  |  | Local media ranked 1st/2nd (info source) → concern (pooled) |  | Neg | 1.95 | * |
|  |  | Perceived fairness of siting (agree fair) → concern (pooled) |  | Neg | 4.52 | ** |
|  |  | Town (Kinuso vs. Swan Hills ref) → concern (pooled) |  | Pos | 3.23 | ** |
|  |  | Outside media ranked 1st/2nd → concern (Swan Hills) |  | Pos | 3.39 | * |
|  |  | Local media ranked 1st/2nd → concern (Fort Assiniboine) |  | Pos | 2.94 | * |
|  |  | Trust in outside media → concern (Fort Assiniboine) |  | Pos | 2.65 | * |
|  |  | Trust in environmental groups → concern (Fort Assiniboine) |  | Pos | 9.47 | ** |
|  |  | Perceived fairness of siting (agree fair) → concern (Fort Assiniboine) |  | Neg | 9.17 | ** |
|  |  | Children →18 at home (yes) → concern (Fort Assiniboine) |  | Neg | 3.27 | * |
|  |  | Trust in environmental groups → concern (Kinuso) |  | Pos | 13.8 | ** |
|  |  | Perceived fairness of siting (agree fair) → concern (Kinuso) |  | Neg | 10.3 | * |
| Besley et al. (2011) | Cancer cluster risk communication & public meetings | Gender (male) → intention to attend future meetings | β | Neg | -0.71 | * |
|  |  | Age → views on public meetings |  | n.s. | 0.00 | n.s. |
|  |  | Age → intention to attend future meetings |  | Neg | -0.01 | *** |
|  |  | Education → views on public meetings |  | Neg | -0.21 | ** |
|  |  | Education → intention to attend future meetings |  | Pos | 0.05 | * |
|  |  | Race (white) → views on public meetings |  | Neg | -1.69 | *** |
|  |  | Race (white) → intention to attend future meetings |  | Neg | -0.07 | n.s. |
|  |  | Attended CC meeting → views on public meetings |  | Pos | 0.64 | # |
|  |  | Attended CC meeting → intention to attend future meetings |  | Pos | 0.47 | *** |
|  |  | Attended other CC meetings → views on public meetings |  | Neg | -0.23 | n.s. |
|  |  | Attended other CC meetings → intention to attend future meetings |  | Pos | 0.35 | ** |
|  |  | Community importance → views on public meetings |  | Pos | 0.40 | *** |
|  |  | Community importance → intention to attend future meetings |  | Pos | 0.04 | *** |
|  |  | Health department credibility → views on public meetings |  | Pos | 0.28 | *** |
|  |  | Health department credibility → intention to attend future meetings |  | Pos | 0.02 | n.s. |
|  |  | Information from health department → views on public meetings |  | Neg | -0.14 | n.s. |
|  |  | Information from health department → intention to attend future meetings |  | Neg | -0.02 | n.s. |
|  |  | Citizen group credibility → views on public meetings |  | Pos | 0.16 | ** |
|  |  | Citizen group credibility → intention to attend future meetings |  | Neg | -0.01 | n.s. |
|  |  | Information from citizen group → views on public meetings |  | Pos | 0.27 | # |
|  |  | Information from citizen group → intention to attend future meetings |  | n.s. | 0.00 | n.s. |
|  |  | Media credibility → views on public meetings |  | Pos | 0.01 | n.s. |
|  |  | Media credibility → intention to attend future meetings |  | Neg | -0.03 | ** |
|  |  | Information from media → views on public meetings |  | Neg | -0.26 | # |
|  |  | Information from media → intention to attend future meetings |  | Pos | 0.01 | n.s. |
|  |  | Cancer risk perception → views on public meetings |  | Pos | 0.07 | n.s. |
|  |  | Cancer risk perception → intention to attend future meetings |  | Pos | 0.01 | n.s. |
|  |  | Views about public meetings → intention to attend future meetings |  | Pos | 0.05 | *** |
|  |  | Identity resilience → fear of COVID-19 |  | Neg | -0.18 | ** |
| Breakwell & Jaspal (2021) | COVID-19 | Fear of COVID-19 → perceived own risk of COVID-19 | β  β | Pos | 0.70 | *** |
|  |  | Trust in science & scientists → perceived own risk of COVID-19 |  | Pos | 0.22 | *** |
|  |  | Identity resilience → uncertainty |  | Neg | -0.14 | * |
|  |  | Fear of COVID-19 → feeling afraid (momentary) |  | Pos | 0.44 | *** |
|  |  | Feeling afraid → identity change |  | Pos | 0.3 | *** |
|  |  | Uncertainty → identity change |  | Pos | 0.16 | * |
|  |  | Uncertainty → mistrust |  | Pos | 0.36 | *** |
|  |  | Mistrust → identity change |  | Pos | 0.15 | * |
|  |  | Trust in science & scientists → mistrust |  | Neg | -0.42 | *** |
|  |  | Fear of COVID-19 → identity change |  | Pos | 0.20 | n.s. |
|  |  | Perceived trust → customer intention to adopt |  | Pos | 0.41 | *** |
| Chawla et al. (2023) | Post COVID-19 FinTech adoption | Perceived security → customer intention to adopt | β  β | Pos | 0.21 | *** |
|  |  | Perceived risk → customer intention to adopt |  | Pos | 0.44 | *** |
|  |  | Perceived security → perceived trust → customer intention to adopt |  | Pos | 0.16 | *** |
|  |  | Perceived risk → perceived trust → customer intention to adopt |  | Pos | 0.22 | *** |
|  |  | Engagement in science communication → intention to correct misinformation (source-based strategies) |  | Pos | 3.53 | *** |
| Choi et al. (2023) | Scientists’ misinformation correction | Engagement in science communication → intention to correct misinformation (relational strategies) | β  β | Pos | 5.47 | *** |
|  |  | Engagement in science communication → attitude toward misinformation |  | Pos | 0.1 | * |
|  |  | Predictors of trust → trust in project manager |  | Pos | 0.8 | *** |
| Conchie & Burns (2009) | Occupational safety in the UK construction industry | Predictors of trust → trust in safety manager | β  β | Pos | 0.47 | *** |
|  |  | Predictors of trust → trust in supervisor |  | Pos | 0.58 | *** |
|  |  | Predictors of trust → trust in UK HSE |  | Pos | 0.59 | *** |
|  |  | Predictors of trust → trust in workmates - accuracy |  | Pos | 0.49 | *** |
|  |  | Predictors of trust → trust in workmates - honesty |  | Pos | 0.21 | * |
|  |  | Trust → intention to change behavior (project manager) |  | Pos | 0.51 | *** |
|  |  | Trust → intention to change behavior (safety manager) |  | Pos | 0.24 | * |
|  |  | Trust → intention to change behavior (workmates) |  | Pos | 0.19 | * |
|  |  | Trust → intention to change behavior (UK HSE) |  | Pos | 0.23 | ** |
|  |  | Trust → intention to change behavior (supervisor) |  | Pos | 0.26 | ** |
|  |  | Perceived ease of use (PEOU) → credibility |  | Pos | 0.35 | *** |
| Corritore et al. (2012) | Online trust in health information websites | PEOU → trust | β  β | Pos | 0.25 | *** |
|  |  | PEOU → risk |  | Neg | -0.05 | n.s. |
|  |  | Credibility → trust |  | Pos | 0.72 | *** |
|  |  | Credibility → risk |  | Neg | -0.48 | *** |
|  |  | Risk → trust |  | Neg | -0.55 | *** |
|  |  | Ideology → populist attitude |  | Neg | -0.2 | *** |
| Erisen & Erisen (2025) | Populism, misinformation, post COVID-19 | Populist attitude → trust in experts | β  SEM | Neg | -0.17 | *** |
|  |  | Populist attitude → misinformation |  | Pos | 0.12 | *** |
|  |  | Populist attitude → trust in institutions |  | Neg | -0.09 | *** |
|  |  | Social media use → misinformation |  | Pos | 0.09 | ** |
|  |  | Misinformation → trust in experts |  | Neg | -0.12 | *** |
|  |  | Misinformation → trust in scientists |  | Neg | -0.08 | * |
|  |  | Misinformation → trust in institutions |  | Neg | -0.09 | ** |
|  |  | Prior attitudes → perceived benefit |  | Pos | 0.92-0.95 | *** |
| Frewer et al. (2003) | Genetically modified (GM) foods | Prior attitudes → perceived risk | SEM  β | Pos | 0.86-0.90 | *** |
|  |  | Information source (industry vs. consumer vs. government) → trust (expertise/trustworthiness) |  | Pos | 0.87-0.90 | *** |
|  |  | Trust (expertise/trustworthiness) → attitudes (benefit/risk) |  | Pos | 0.01 | ** |
|  |  | (2020 survey) Age of 60s → disagree to restart |  | Pos | 1.15 | ** |
| Fujita (2025) | Nuclear power (post-Fukushima) | Self-employment → agree to restart | β  β | Neg | -0.92 | * |
|  |  | Trust in nuclear operators → agree to restart |  | Pos | 1.31 | ** |
|  |  | Trust in nuclear regulators → disagree to restart |  | Neg | -1.28 | ** |
|  |  | Benefit perception → agree to restart |  | Pos | 0.40 | *** |
|  |  | Benefit perception → disagree to restart |  | Neg | -0.43 | ** |
|  |  | Risk perception → agree to restart |  | Neg | -0.48 | *** |
|  |  | Subjective knowledge → agree to restart |  | Pos | 1.18 | ** |
|  |  | Objective knowledge → agree to restart |  | Pos | 0.21 | *** |
|  |  | (2021 survey) Male → agree to restart |  | Pos | 0.68 | * |
|  |  | Conservative identification → disagree to restart |  | Neg | -0.64 | * |
|  |  | Trust in nuclear operators → agree to restart |  | Pos | 0.81 | * |
|  |  | Trust in nuclear operators → disagree to restart |  | Neg | -1.14 | ** |
|  |  | Trust in nuclear regulators → agree to restart |  | Pos | 0.74 | * |
|  |  | Trust in nuclear regulators → disagree to restart |  | Neg | -1.15 | * |
|  |  | Benefit perception → agree to restart |  | Pos | 0.47 | *** |
|  |  | Benefit perception → disagree to restart |  | Neg | -0.63 | *** |
|  |  | Risk perception → agree to restart |  | Neg | -0.51 | *** |
|  |  | Risk perception → disagree to restart |  | Pos | 0.47 | *** |
|  |  | Subjective knowledge → agree to restart |  | Pos | 1.24 | ** |
|  |  | Subjective knowledge → disagree to restart |  | Pos | 1.34 | ** |
|  |  | Objective knowledge → agree to restart |  | Pos | 0.17 | ** |
|  |  | Information seeking → media distort reality (belief) |  | Neg | -0.38 | *** |
| Guo & Bai (2023) | COVID-19 | Media distort reality → fear | β  β | Pos | 0.08 | ** |
|  |  | Indirect effect: Information seeking → media distort reality → fear |  | Neg | -0.03 | 95% CI [�0.0534, �0.0112] |
|  |  | Information seeking → media provide validity cues (belief) |  | Pos | 0.19 | *** |
|  |  | Media provide validity cues → calmness |  | Pos | 0.12 | *** |
|  |  | Indirect effect: Information seeking → media provide validity cues → calmness |  | Pos | 0.02 | 95% CI [0.0115, 0.0368] |
|  |  | Information seeking � Heuristic processing → media distort reality |  | Neg | -0.09 | *** |
|  |  | Information seeking � Systematic processing → media provide validity cues |  | Neg | -0.08 | *** |
|  |  | Content credibility → adoption willingness |  | Pos | 0.23 | *** |
| Jin et al. (2021) | Health knowledge communication on social media | Source credibility → sharing willingness | β  β | Pos | 0.34 | *** |
|  |  | Source credibility → adoption willingness |  | Pos | 0.54 | *** |
|  |  | Institution-based trust → sharing willingness |  | Pos | 0.46 | *** |
|  |  | Institution-based trust trust → adoption willingness |  | Pos | 0.18 | *** |
|  |  | (All respondents) Perception of efficiency → acceptability |  | Pos | 0.41 | ** |
| Katsuya (2002) | Nuclear power | Perception of sufficiency of electric power → acceptability | β  β | Pos | 0.08 | ** |
|  |  | Risk perception → acceptability |  | Neg | -0.23 | ** |
|  |  | Trust in nuclear power operation → acceptability |  | Pos | 0.28 | ** |
|  |  | (High motivation group) Perception of efficiency → acceptability |  | Pos | 0.28 | ** |
|  |  | Perception of sufficiency of electric power → acceptability |  | Pos | 0.11 | ** |
|  |  | Risk perception → acceptability |  | Neg | -0.38 | ** |
|  |  | Trust in nuclear power operation → acceptability |  | Pos | 0.20 | * |
|  |  | (Low motivation group) Perception of efficiency → acceptability |  | Pos | 0.38 | ** |
|  |  | Trust in nuclear power operation → acceptability |  | Pos | 0.54 | ** |
|  |  | (Extremely high motivation group) Perception of efficiency → acceptability |  | Pos | 0.33 | ** |
|  |  | Perception of sufficiency of electric power → acceptability |  | Pos | 0.13 | * |
|  |  | Risk perception → acceptability |  | Neg | -0.26 | * |
|  |  | Trust in nuclear power operation → acceptability |  | Pos | 0.26 | * |
|  |  | (Extremely low motivation group) Perception of efficiency → acceptability |  | Pos | 0.37 | ** |
|  |  | Trust in nuclear power operation → acceptability |  | Pos | 0.50 | ** |
|  |  | Need for cognition (NFC) → trust in selectivity of facts (NTselectivity) |  | Neg | -0.12 | * |
| Kožuh & Čakš (2021) | COVID-19 | News engagement (prior knowledge) → NTselectivity | β  OR | Pos | 0.21 | *** |
|  |  | News engagement (prior knowledge) → news engagement (interest) |  | Pos | 0.24 | *** |
|  |  | NTselectivity → trust in accuracy of depictions (NTaccuracy) |  | Pos | 0.56 | *** |
|  |  | News engagement (interest) → NTaccuracy |  | Pos | 0.15 | * |
|  |  | Trust in mainstream sources (MS) → Belief vaccine prevents infection |  | Pos | 1.47 | ** |
| Latkin et al. (2021) | COVID-19 | Trust in MS → Encourage family to vaccinate | OR  β | Pos | 1.61 | *** |
|  |  | Trust in MS → Altruistic beliefs (elderly priority) |  | Pos | 1.50 | ** |
|  |  | Trust in MS → Altruistic beliefs (minority priority) |  | Pos | 1.32 | ** |
|  |  | Trust in PCS (Fox/White House) → Discourage friends to vaccinate |  | Pos | 1.47 | ** |
|  |  | Trust in PCS → Altruistic beliefs (minority priority) |  | Pos | 0.70 | ** |
|  |  | Trust in Dr. Fauci → Belief vaccine prevents infection |  | Pos | 1.49 | ** |
|  |  | Trust in Dr. Fauci → Encourage family to vaccinate |  | Pos | 1.68 | *** |
|  |  | Trust in Dr. Fauci → Discourage friends to vaccinate |  | Pos | 0.67 | ** |
|  |  | Political affiliation (Democrat) → Trust in MS |  | Pos | 2.82 | ** |
|  |  | Political affiliation (Democrat) → Trust in Dr. Fauci |  | Pos | 4.23 | *** |
|  |  | Political affiliation (Democrat) → Trust in PCS |  | Pos | 0.11 | *** |
|  |  | (Scientific information) Argument quality (ARQ) → perceived credibility (INC) |  | Pos | 0.18 | ** |
| Lederman et al. (2014) | Online health forums | Verification (VER) → INC | β  β | Pos | 0.40 | **** |
|  |  | Literacy competence (LIC) → INC |  | Pos | 0.18 | * |
|  |  | Reference credibility (REC) → INC |  | Pos | 0.22 | **** |
|  |  | (Experiential information) ARQ → INC |  | Pos | 0.18 | ** |
|  |  | VER → INC |  | Pos | 0.36 | **** |
|  |  | LIC → INC |  | Pos | 0.21 | *** |
|  |  | Crowd consensus (CRC) → INC |  | Pos | 0.25 | ** |
|  |  | Education → belief in News A |  | Pos | 0.12 | * |
| Lee & Jeong (2024) | COVID-19 | Political orientation → belief in News A | β  β | Neg | -0.17 | *** |
|  |  | Risk perception → belief in News A |  | Pos | 0.10 | * |
|  |  | Risk perception → general social distrust |  | Pos | 0.23 | *** |
|  |  | Belief in News A → general social distrust |  | Pos | 0.24 | *** |
|  |  | Education → belief in News B |  | Neg | -0.12 | ** |
|  |  | Political orientation → belief in News B |  | Neg | -0.12 | ** |
|  |  | Risk perception → belief in News B |  | Pos | 0.11 | ** |
|  |  | Risk perception → general social distrust |  | Pos | 0.16 | *** |
|  |  | Belief in News B → general social distrust |  | Pos | 0.11 | *** |
|  |  | AI-assisted text → Perceived clarity of scientific explanations |  | Pos | 0.32 | *** |
| Markowitz (2024) | AI & science communication | AI-assisted text → Perceived competence of scientists | β  β | Pos | 0.27 | *** |
|  |  | AI-assisted text → Trust in scientists |  | Pos | 0.22 | ** |
|  |  | AI-assisted text → Public understanding of science |  | Pos | 0.35 | *** |
|  |  | Complexity of original scientific text → Perceived clarity (w/o AI) |  | Neg | -0.41 | *** |
|  |  | Complexity � AI presence → Perceived clarity |  | Pos | 0.18 | ** |
|  |  | AI use → Concerns about authenticity |  | Pos | 0.14 | * |
|  |  | Female → use of new media |  | Pos | 0.21 | * |
| Myrick & Hendryx (2021) | Rural health disparities (Appalachia, mining vs. non-mining communities) | Female → use of friends | β  β | Pos | 0.24 | * |
|  |  | Age → use of nurses |  | Neg | -0.58 | *** |
|  |  | Symptoms → use of nurses |  | Pos | 0.30 | * |
|  |  | Smoker → use of nurses |  | Neg | -0.24 | * |
|  |  | Miner → use of nurses |  | Pos | 0.21 | * |
|  |  | Symptoms → use of doctors |  | Pos | 0.37 | ** |
|  |  | White → use of health department |  | Neg | -0.4 | *** |
|  |  | Education → trust in friends and family |  | Pos | 0.26 | * |
|  |  | White → trust in friends and family |  | Pos | 0.29 | ** |
|  |  | Mining community → trust in friends and family |  | Pos | 0.31 | ** |
|  |  | Determinants of trust (Voluntary condition): Competency |  | Pos | 0.57 | *** |
| Nakayachi & Ozaki (2014) | Disaster risk management (Fukushima nuclear accident, Japan) | Determinants of trust (Voluntary condition): Motivation | β  t-test | Pos | 0.35 | *** |
|  |  | Trust in health orgs → government health agencies (general health info) |  | Pos | 3.96 | *** |
| Peterson et al. (2020) | Health information sources about tobacco | Trust in health orgs → government health agencies (tobacco info) | t-test  OR | Pos | 5.17 | *** |
|  |  | Trust in health orgs → government health agencies (e-cigarette info) |  | Pos | 5.59 | *** |
|  |  | Confidence in obtaining tobacco health info → trust in government agencies (general health info) |  | Pos | 0.3 | *** |
|  |  | Confidence in obtaining tobacco health info → trust in government agencies (tobacco info) | OR  β | Pos | 0.26 | *** |
|  |  | Confidence in obtaining tobacco health info → trust in government agencies (e-cigarette info) |  | Pos | 0.18 | *** |
|  |  | Confidence in obtaining tobacco health info → trust in health orgs (general health info) |  | Pos | 0.31 | *** |
|  |  | Confidence in obtaining tobacco health info → trust in health orgs (tobacco info) |  | Pos | 0.30 | *** |
|  |  | Confidence in obtaining tobacco health info → trust in health orgs (e-cigarette info) |  | Pos | 0.25 | *** |
|  |  | Source credibility → trust in government |  | Pos | 0.53 | *** |
| Ryu et al. (2018) | Nuclear power | Source credibility → perceived risk of nuclear power energy | β  β | Neg | -0.08 | n.s. |
|  |  | Source credibility → trust in regulation |  | Pos | 0.62 | *** |
|  |  | Trust in government → trust in regulation |  | Pos | 0.10 | *** |
|  |  | Trust in government → acceptability of nuclear power energy |  | Pos | 0.17 | *** |
|  |  | Trust in government → perceived risk of nuclear power energy |  | Neg | -0.15 | *** |
|  |  | Trust in regulation → perceived risk of nuclear power energy |  | Neg | -0.03 | *** |
|  |  | Trust in regulation → acceptability of nuclear power energy |  | Pos | 0.31 | *** |
|  |  | Perceived risk of nuclear power energy → acceptability of nuclear power energy |  | Neg | -0.28 | *** |
|  |  | Trust in local government → flood risk perception (municipality) |  | Neg | -0.41 | ** |
| Seebauer & Babcicky (2017) | Flood risks | Trust in local government → flood risk perception (own building) | β  β | Neg | -0.33 | ** |
|  |  | Trust in local government → flood risk perception (fear) |  | Neg | -0.22 | ** |
|  |  | Trust in volunteers → flood risk perception (municipality) |  | Pos | 0.16 | ** |
|  |  | Trust in volunteers → flood risk perception (own building) |  | Pos | 0.09 | ** |
|  |  | Trust in local government → intention of implementing private measures |  | Neg | -0.06 | * |
|  |  | Trust in local government → reliance on social support |  | Pos | 0.2 | ** |
|  |  | Trust in local government → reliance on public protection |  | Pos | 0.81 | ** |
|  |  | Trust in local government → fatalism |  | Pos | 0.27 | ** |
|  |  | Trust in local government → denial |  | Pos | 0.19 | ** |
|  |  | Trust in local government → wishful thinking |  | Pos | 0.37 | ** |
|  |  | Trust in volunteers → intention of implementing private measures |  | Pos | 0.09 | ** |
|  |  | Trust in volunteers → reliance on social support |  | Pos | 0.13 | ** |
|  |  | Trust in volunteers → denial |  | Neg | -0.13 | ** |
|  |  | Trust in volunteers → wishful thinking |  | Pos | 0.1 | ** |
|  |  | Trust in neighbors → intention of implementing private measures |  | Pos | 0.13 | ** |
|  |  | Trust in neighbors → reliance on social support |  | Pos | 0.31 | ** |
|  |  | Trust in neighbors → wishful thinking |  | Pos | 0.10 | ** |
|  |  | (Pesticides) Value similarity → trust |  | Pos | 0.64 | * |
| Siegrist et al. (2000) | Technological & environmental risks (pesticides, nuclear power, artificial sweetener) | Trust → perceived benefit | β  Mean Diff. | Pos | 0.78 | * |
|  |  | Trust → perceived risk |  | Neg | -0.77 | * |
|  |  | (Nuclear power) Value similarity → trust |  | Pos | 0.65 | * |
|  |  | Trust → Perceived benefit |  | Pos | 0.72 | * |
|  |  | Trust → perceived risk |  | Neg | -0.84 | * |
|  |  | (Artificial sweetener) Value similarity → trust |  | Pos | 0.58 | * |
|  |  | Trust → perceived benefit |  | Pos | 0.54 | * |
|  |  | Trust → perceived risk |  | Neg | -0.81 | * |
|  |  | (Cognitive trust) Doctor vs. WebMD |  | Pos | 1.29 | * |
| Song et al. (2024) | Health information seeking & misinformation | Doctor vs. Wikipedia | Mean Diff.  Odds Ratio | Pos | 1.53 | * |
|  |  | Close friend/family vs. WebMD |  | Pos | 1.21 | * |
|  |  | Close friend/family vs. Wikipedia |  | Pos | 1.46 | * |
|  |  | (Behavioral trust) WebMD (compared to reference) |  | Pos | 0.04 | ** |
|  |  | Wikipedia (compared to reference) | Odds Ratio  Mean Diff. | Pos | 0.02 | ** |
|  |  | Friend/family member (compared to reference) |  | Pos | 0.03 | ** |
|  |  | Symptom intensity (low vs. high) → cognitive trust |  | Pos | 0.20 | * |
|  |  | Disease type (acute vs. chronic) → cognitive trust | Mean Diff.  Corr. | Pos | 0.06 | n.s. |
|  |  | Information sufficiency → Affective responses |  | Neg | -0.36 | *** |
| Huurne & Gutteling (2009) | Hazardous industrial chemicals | Information sufficiency → Institutional trust | Corr.  β | Pos | 0.29 | *** |
|  |  | Information sufficiency → Self-efficacy |  | Pos | 0.64 | *** |
|  |  | Affective responses → Institutional trust |  | Neg | -0.29 | *** |
|  |  | Affective responses → Self-efficacy |  | Neg | -0.34 | *** |
|  |  | Institutional trust → Self-efficacy |  | Pos | 0.27 | *** |
|  |  | Gender (female vs. male) → Preventive behaviors |  | Neg | -0.68 | *** |
| Tetteh et al. (2022) | COVID-19 | Age (46-65 vs. 18-45) → Preventive behaviors | β  β | Pos | 0.03 | * |
|  |  | Income (US $40-70k vs. <40k) → Preventive behaviors |  | Pos | 0.04 | * |
|  |  | Employment (work from home) → Preventive behaviors |  | Pos | 0.05 | *** |
|  |  | Employment (not working) → Preventive behaviors |  | Pos | 0.05 | * |
|  |  | County (St. Louis County vs. City) → Preventive behaviors |  | Neg | -0.02 | * |
|  |  | Vulnerable health conditions → Preventive behaviors |  | Pos | 0.05 | * |
|  |  | Trust in Public Health Agencies → Preventive behaviors |  | Pos | 0.12 | * |
|  |  | Trust � Gender (men) → Preventive behaviors |  | Pos | 0.52 | *** |
|  |  | Week of data collection → Preventive behaviors |  | Pos | 0.02 | *** |
|  |  | Citizen credibility → risk perception (total) |  | Pos | 0.28 | * |
| Trumbo & McComas (2003) | Cancer cluster investigations | State credibility → risk perception (total) | β  β | Neg | -0.26 | * |
|  |  | Industrial credibility → risk perception (total) |  | Neg | -0.19 | * |
|  |  | Citizen credibility → risk perception (direct) |  | Pos | 0.23 | * |
|  |  | State credibility → risk perception (direct) |  | Neg | -0.18 | * |
|  |  | Industrial credibility → risk perception (direct) |  | Neg | -0.13 | * |
|  |  | Citizen credibility → risk perception (indirect) |  | Pos | 0.05 | * |
|  |  | State credibility → risk perception (indirect) |  | Neg | -0.08 | * |
|  |  | Industrial credibility → risk perception (indirect) |  | Neg | -0.06 | * |
|  |  | Perceived nuclear risk → nuclear acceptance |  | Neg | -0.58 | *** |
| Vainio et al. (2016) | Nuclear power (climate change mitigation) | Perceived nuclear risk → WTP for alternatives | β  β | Pos | 0.54 | *** |
|  |  | Climate change concern → WTP for alternatives |  | Pos | 0.36 | *** |
|  |  | Trust in nuclear companies → perceived nuclear risk |  | Neg | -0.34 | *** |
|  |  | Trust in nuclear companies → nuclear acceptance |  | Pos | 0.41 | *** |
|  |  | Trust in nuclear companies → WTP for alternatives |  | Neg | -0.22 | *** |
|  |  | Trust in NGOs → perceived nuclear risk |  | Pos | 0.45 | *** |
|  |  | Trust in NGOs → nuclear acceptance |  | Neg | -0.38 | *** |
|  |  | Trust in NGOs → WTP for alternatives |  | Pos | 0.38 | *** |
|  |  | Trust in public institutions → nuclear acceptance |  | Pos | 0.44 | *** |
|  |  | Trust in public institutions → WTP for alternatives |  | Pos | 0.25 | *** |
|  |  | Attitude - RIA |  | Pos | 0.82 | *** |
| Wang et al. (2022) | COVID-19 (risk information avoidance) | Risk perception - Affective risk response | β  β | Pos | 0.31 | *** |
|  |  | Affect � RIA |  | Neg | -0.04 | n.s. |
|  |  | Untrustworthiness - Risk perception |  | Pos | 0.06 | n.s. |
|  |  | Untrustworthiness - Attitude |  | Pos | 0.53 | *** |
|  |  | Untrustworthiness - Norm |  | Pos | 0.49 | *** |
|  |  | SDO - Norm |  | Pos | 0.37 | *** |
|  |  | SDO - Untrustworthiness |  | Pos | 0.56 | *** |
|  |  | SDO - Risk perception |  | Pos | 0.17 | ** |
|  |  | SDO - Attitude |  | Pos | 0.33 | *** |
|  |  | Norm - RIA |  | Pos | 0.19 | *** |
|  |  | Ability → trust |  | Pos | 0.2 | ** |
| White & Johnson (2010) | Bird flu | Bias → trust | β  β | Pos | 0.4 | *** |
|  |  | Openness → trust |  | Pos | 0.41 | *** |
|  |  | Bias → trust |  | Pos | 0.5 | *** |
|  | IT virus | Openness → trust |  | Pos | 0.48 | *** |
|  |  | Bias → trust |  | Pos | 0.14 | * |
|  | Terror | Ability → trust |  | Pos | 0.42 | *** |
|  | Water | Openness → trust |  | Pos | 0.39 | *** |
|  |  | Bias → trust |  | Pos | 0.15 | ** |
|  | Air | Openness → trust |  | Pos | 0.49 | *** |
|  |  | Ability → trust |  | Pos | 0.24 | *** |
|  | Cancer | Bias → trust |  | Pos | 0.11 | * |
|  |  | Openness → trust |  | Pos | 0.59 | *** |
|  |  | Ability → trust |  | Pos | 0.26 | *** |
|  | Brownfields | Openness → trust |  | Pos | 0.54 | *** |
|  |  | Age → online channel preference |  | Pos | 0.16 | *** |
| Wilkins et al. (2018) | Environmental conservation | Age → visual media preference | β  GLMM β | Pos | 0.08 | * |
|  |  | Age → printed media preference |  | Pos | 0.11 | ** |
|  |  | Age → personal experience/ communication preference |  | Neg | -0.12 | ** |
|  |  | Gender → educational opportunities preference |  | Pos | 0.07 | * |
|  |  | Education → online channel preference |  | Pos | 0.18 | *** |
|  |  | Education → visual media preference |  | Pos | 0.07 | * |
|  |  | Education → audio channel preference |  | Pos | 0.08 | * |
|  |  | Education → printed media preference |  | Pos | 0.08 | * |
|  |  | Education → personal experience/ communication preference |  | Pos | 0.14 | *** |
|  |  | Education → educational opportunities preference |  | Pos | 0.19 | *** |
|  |  | Childhood residence → printed media preference |  | Neg | -0.13 | *** |
|  |  | Childhood residence → personal experience/ communication preference |  | Neg | -0.13 | *** |
|  |  | Race → online channel preference |  | Neg | -0.08 | * |
|  |  | Race → personal experience/ communication preference |  | Neg | -0.09 | * |
|  |  | Age → trust in government |  | Pos | 0.07 | * |
|  |  | Age → trust in news |  | Pos | 0.15 | *** |
|  |  | Age → trust in religion |  | Pos | 0.19 | *** |
|  |  | Gender → trust in scientific organizations |  | Pos | 0.17 | *** |
|  |  | Gender → trust in news |  | Pos | 0.16 | *** |
|  |  | Education → trust in government |  | Pos | 0.18 | *** |
|  |  | Education → trust in scientific organizations |  | Pos | 0.21 | *** |
|  |  | Education → trust in news |  | Pos | 0.13 | *** |
|  |  | Education → trust in religion |  | Neg | -0.07 | * |
|  |  | Current residence → trust in government |  | Pos | 0.10 | ** |
|  |  | Current residence → trust in scientific organizations |  | Pos | 0.08 | * |
|  |  | Current residence → trust in news |  | Pos | 0.09 | * |
|  |  | Childhood residence → trust in family/ friends |  | Neg | -0.08 | * |
|  |  | Race → trust in scientific organizations |  | Neg | -0.08 | * |
|  |  | Race → trust in news |  | Pos | 0.089 | * |
|  |  | Race → trust in family/ friends |  | Neg | -0.12 | ** |
|  |  | Race → trust in religion |  | Pos | 0.08 | ** |
|  |  | Ethnicity → trust in government |  | Neg | -0.08 | * |
|  |  | Ethnicity → trust in religion |  | Neg | -0.10 | *** |
|  |  | Age (26�30) → Trust in social media |  | Pos | 0.49 | ** |
| Wilson et al. (2016) | Science communication (trust in sources) | Non-academic scientists → Trust in social media | GLMM β  β | Neg | -0.43 | * |
|  |  | General public → Trust in social media |  | Neg | -0.42 | *** |
|  |  | General public → Trust in journals |  | Neg | -0.37 | *** |
|  |  | General public → Trust in personal experience |  | Neg | -0.35 | ** |
|  |  | General public → Use of social media |  | Neg | -1.25 | *** |
|  | Science communication (use of sources) | Policymakers → Use of social media |  | Neg | -0.88 | * |
|  |  | Students → Use of journals |  | Neg | -0.56 | *** |
|  |  | Age (51�55) → Use of personal experience |  | Neg | -0.58 | * |
|  |  | Age (56�60) → Use of personal experience |  | Neg | -0.81 | ** |
|  |  | Age (61�65) → Use of personal experience |  | Neg | -1.01 | *** |
|  |  | Age (66+) → Use of personal experience |  | Neg | -0.87 | ** |
|  |  | Argument quality → perceived information quality |  | Pos | 0.36 | *** |
| Yi et al. (2013) | Web-based health information | Argument quality → perceived risk | β  β | Pos | 0.06 | n.s. |
|  |  | Source expertise → perceived information quality |  | Pos | 0.26 | ** |
|  |  | Source expertise → perceived risk |  | Neg | -0.11 | n.s. |
|  |  | Perceived information quality → perceived risk |  | Neg | -0.47 | *** |
|  |  | perceived information quality → trust |  | Pos | 0.74 | *** |
|  |  | Perceived risk → trust |  | Neg | -0.17 | * |
|  |  | PR → TNS (combined) |  | Neg | -0.58 | *** |
| Zhang et al. (2025) | AI-generated misinformation & deepfakes | PR → ISB (combined) | β  β | Pos | 0.17 | *** |
|  |  | ISB → EDF (combined) |  | Pos | 0.64 | *** |
|  |  | PR → ISB → TNS (combined) |  | Neg | -0.02 | *** |
|  |  | ISB → EDF → TNS (combined) |  | Neg | -0.11 | *** |
|  |  | PR → ISB → EDF → TNS (combined) |  | Neg | -0.02 | *** |
|  |  | PR x EAI → ISB (combined) |  | Pos | 0.07 | *** |
|  |  | ISB x EAO → EDF (combined) |  | Pos | 0.09 | *** |
|  |  | Perceived Knowledge → Information Insufficiency |  | Pos | 0.40 | *** |
| Zhu et al. (2016) | Nuclear power | Perceived Knowledge → Information Seeking | β | Pos | 0.26 | *** |
|  |  | Information Insufficiency → Information Seeking |  | Pos | 0.25 | *** |
|  |  | Perceived Knowledge → Systematic Processing |  | Pos | 0.23 | *** |
|  |  | Information Insufficiency → Systematic Processing |  | Pos | 0.30 | *** |
|  |  | Information Seeking → Systematic Processing |  | Pos | 0.35 | *** |
|  |  | Perceived Knowledge → Risk Perception |  | Neg | -0.14 | * |
|  |  | Systematic Processing → Risk Perception |  | Pos | 0.21 | ** |
|  |  | Risk Perception → Behavioral Intentions (anti-nuclear) |  | Pos | 0.42 | *** |
|  |  | Perceived Knowledge → Behavioral Intentions |  | Neg | -0.18 | ** |
|  |  | Systematic Processing → Behavioral Intentions |  | n.s. | n.s. | n.s. |
|  |  |  |  |  |  |  |

* = p < .05, ** = p < .01, and *** = p < .001; n.s. = not significant.
